# Biomimetic MDSCs membrane coated black phosphorus nanosheets system for photothermal therapy/photodynamic therapy synergized chemotherapy of cancer

**DOI:** 10.1186/s12951-024-02417-4

**Published:** 2024-04-12

**Authors:** Zhou Lan, Wei-Jia Liu, Wu-Wei Yin, Sheng-Ren Yang, Hao Cui, Ke-Long Zou, Guo-Wang Cheng, Hao Chen, Yan-Hua Han, Lang Rao, Rui Tian, Ling-Ling Li, Yu-Yue Zhao, Guang-Tao Yu

**Affiliations:** 1 Stomatological Hospital, School of Stomatology, Southern Medical University, No 366, Jiangnan Road, Haizhu Region, Guangzhou City, China; 2 Department of Oral Mucosal Diseases, Guangdong Engineering Research Center of Oral Restoration and Reconstruction, Guangzhou Key Laboratory of Basic and Applied Research of Oral Regenerative Medicine, Affiliated Stomatology Hospital of Guangzhou Medical University, Guangzhou, Guangdong 510182 China; 3 State Key Laboratory of Molecular Vaccinology and Molecular Diagnostics, Center for Molecular Imaging and Translational Medicine, School of Public Health, Xiamen University, Xiamen, 361102 China; 4 Science and Technology Innovation Center, Guangzhou University of Chinese Medicine, Guangzhou, 510405 China; 5 Institute of Biomedical Health Technology and Engineering, Shenzhen Bay Laboratory, Shenzhen, 518132 China; 6 Department of Pharmaceutics, School of Pharmacy, Nanjing Medical University, No 101, Longmian Road, Jiangning Region, Nanjing, 211166 China

**Keywords:** Oral squamous cell carcinoma, Black phosphorous, Decitabine, MDSCs, Photothermal therapy, Photodynamic therapy, Chemotherapy, Immunotherapy

## Abstract

Photothermal therapy is favored by cancer researchers due to its advantages such as controllable initiation, direct killing and immune promotion. However, the low enrichment efficiency of photosensitizer in tumor site and the limited effect of single use limits the further development of photothermal therapy. Herein, a photo-responsive multifunctional nanosystem was designed for cancer therapy, in which myeloid-derived suppressor cell (MDSC) membrane vesicle encapsulated decitabine-loaded black phosphorous (BP) nanosheets (BP@ Decitabine @MDSCs, named BDM). The BDM demonstrated excellent biosafety and biochemical characteristics, providing a suitable microenvironment for cancer cell killing. First, the BDM achieves the ability to be highly enriched at tumor sites by inheriting the ability of MDSCs to actively target tumor microenvironment. And then, BP nanosheets achieves hyperthermia and induces mitochondrial damage by its photothermal and photodynamic properties, which enhancing anti-tumor immunity mediated by immunogenic cell death (ICD). Meanwhile, intra-tumoral release of decitabine induced G2/M cell cycle arrest, further promoting tumor cell apoptosis. In vivo, the BMD showed significant inhibition of tumor growth with down-regulation of PCNA expression and increased expression of high mobility group B1 (HMGB1), calreticulin (CRT) and caspase 3. Flow cytometry revealed significantly decreased infiltration of MDSCs and M2-macrophages along with an increased proportion of CD4^+^, CD8^+^ T cells as well as CD103^+^ DCs, suggesting a potentiated anti-tumor immune response. In summary, BDM realizes photothermal therapy/photodynamic therapy synergized chemotherapy for cancer.

**Supplementary Information:**

The online version contains supplementary material available at 10.1186/s12951-024-02417-4.

## Introduction

Photothermal therapy (PTT) and photodynamic therapy (PDT) have been extensively utilized as novel approaches for cancer treatment due to their non-invasive nature and efficient tumor eradication effect [1, 2]. When the photosensitizer aggregates at the tumor site, photosensitizer-induced PDT generates a substantial amount of reactive oxygen species (ROS) to achieve a precise and controllable spatial-temporal tumor killing effect. Meanwhile, photosensitizer-induced PTT induces local thermal damage by converting light energy into thermal energy, facilitating the thermal ablation of tumor tissue [3]. However, conventional photosensitizers or photothermal conversion agents require enhanced permeability and retention (EPR) effect for effective implantation in tumors [4]. Additionally, both therapies are limited by the shallow tissue penetration depth of light, making them more suitable for superficial tumors [5]. Oral squamous cell carcinoma (OSCC), originating from oral mucosal epithelium, is considered a superficial tumor. Current standard treatments consist of surgical resection, adjuvant radiotherapy and chemotherapy [6, 7]. Despite the application of various novel strategies such as immunotherapy [8], targeted therapy, nanomaterial-based PTT [9, 10], and PDT [11] alone, the overall survival rate is still below 50% [12]. Therefore, an effective strategy combining these therapies is needed to overcome notable limitations including low efficiency, limited penetration depth, lack of target specificity, and poor biocompatibility.

In recent years, drug delivery systems based on emerging nanomaterials such as black phosphorus (BP), graphdiyne, selenium, and bismuth have demonstrated significant potential in the field of anti-tumor therapy [13–16]. As an emerging two-dimensional layered nanomaterial, BP has attracted much attention in the field of biomedical research because of its unique physical properties [17]. Its folded lattice structure provides a large surface area, resulting in efficient drug loading capacity [18]. Its unique electronic structure and broad absorption peak in the visible region endow it with strong singlet oxygen generation ability and excellent photothermal performance, yielding extraordinary results in anti-tumor therapy [19]. The hydrogel encapsulated BP and DOX nanodrug delivery system, responsive to near-infrared light, exhibits remarkable efficacy in tumor eradication through PTT and chemotherapy [13]. HA-modified BP showed strong tumor therapeutic potential through PTT, PDT and induced immunogenic cell death (ICD) [20]. Other studies have shown that BP can directly combine with PLK1 to mediate the deactivation of centrosome kinase polo-like kinase 1 (PLK1), affecting mitosis during the cell cycle and promoting tumor cell apoptosis [21]. It can also induce mitochondrial oxidative stress by producing a large amount of reactive oxygen species, thereby interfering with normal cellular functions and inducing apoptosis [22]. Decitabine is different from conventional chemotherapy drugs in that it mainly inhibits the growth of tumor cells by demethylation and inducing G2/M phase cell cycle arrest [23, 24]. At present, decitabine is only used for the treatment of myelodysplastic syndrome (MDS). There are few reports on the treatment effect of solid tumors due to its myelosuppressive toxicity [25].

Myeloid-derived suppressor cells (MDSCs), which have been proven to be a critical part of tumor immune microenvironment (TIME), can be derived not only from myeloid progenitor cells but also immature myeloid cells [26]. The accumulation of MDSCs at tumor site relies on various factors such as chemokines and cytokines produced by tumor cells [27]. It has been found that the recruitment of MDSCs is mediated by highly expressed CCR1, CCR2, CCR5, CCR7, CX3CR1, CXCR1, CXCR2, CXCR5 in MDSCs membranes [28]. In addition, with the development of cell membrane biomimetic nanotechnology, this property can be used to actively target tumor sites accompanied with another anti-tumor therapy. In our previous work, GNRs@SiO2@MnO2 nanoparticles were successfully coated with MDSCs membranes to eliminate cancer by providing immune evasion and superior targeting capacity [29].

**Scheme 1:**
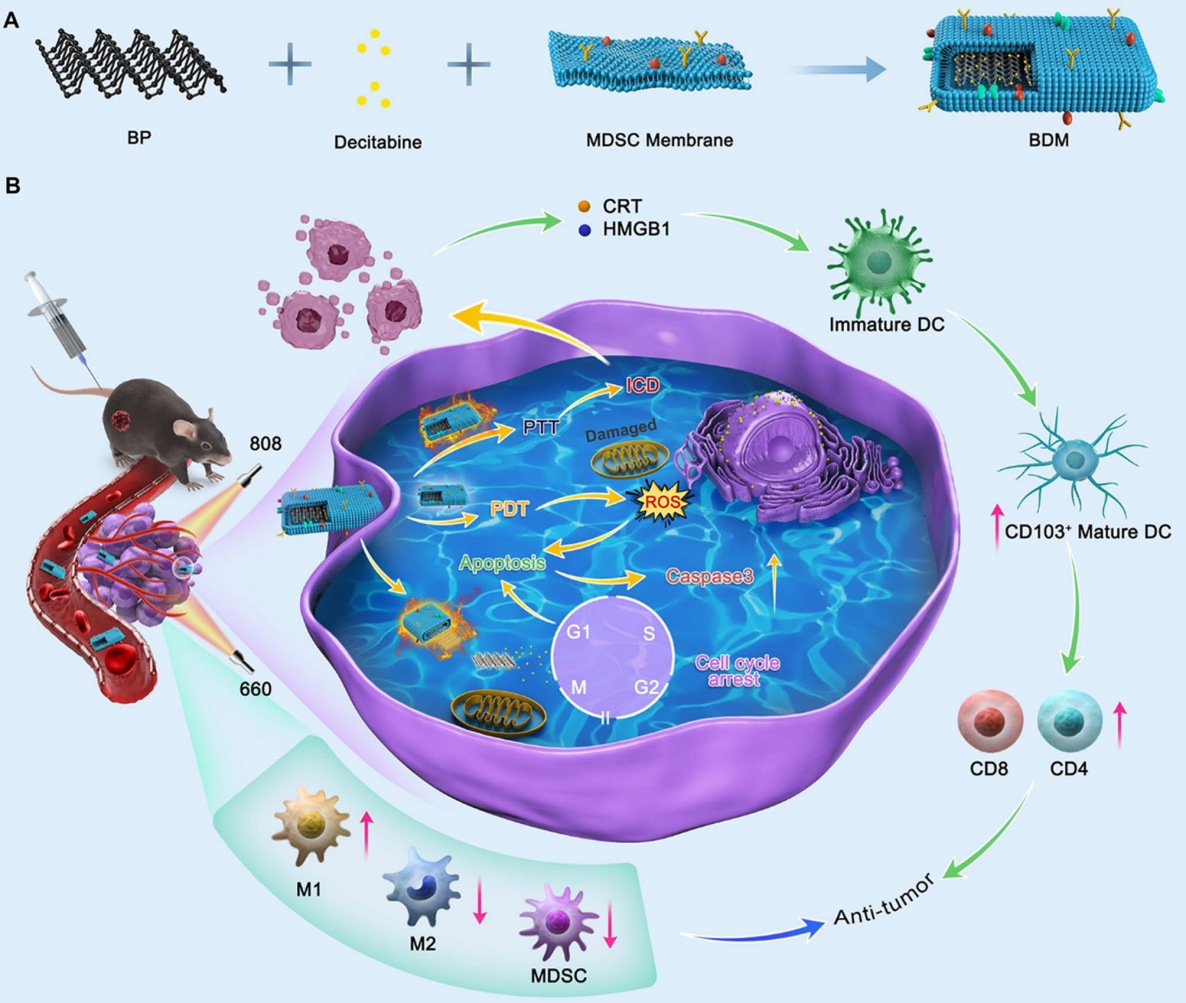
Schematic overview illustrating the preparation and the anti-tumor therapy of BDM. (**A**) Brief schematic of BDM synthesis. (**B**). The anti-tumor mechanism of BDM via PTT, PDT, Apoptosis and ICD induced enhancing anti-tumor immunity. (BP: black phosphorous; BDM: MDSCs membrane coated BP with decitabine loaded; PTT: Photothermal Therapy; PDT: Photodynamic Therapy; ICD: Immunologic cell death)

For more effective cancer treatment, we designed a BP-based drug delivery nanoplatform: myeloid-derived suppressor cells (MDSCs) membrane vesicle encapsulated decitabine-loaded black phosphorous (BP) nanosheets (BP@ Decitabine @MDSCs, named BDM) (Scheme 1). Membrane proteins of MDSCs endow BDM with the potential to actively target tumor tissue and reduce the myelotoxicity of decitabine. Meanwhile, the photothermal and photodynamic activities of BP can not only achieve hyperthermia and ROS-mediated mitochondrial damage, but also promote ICD-mediated anti-tumor immune enhancement. Finally, locally applicated decitabine further induce tumor cell demethylation and cell cycle blockade. A multi-therapeutic model integrating photothermal therapy, photodynamic therapy, chemotherapy and immunotherapy can be realized, providing a promising approach for cancer therapy.

## Materials and methods

### Reagents and machine

Bulk black phosphorous were purchased from xfnano materials tech co., ltd. 1-Methyl-2-pyrrolidinone (NMP, M103246) was purchased from Aladdin. Decitabine (HY-A0004) were purchased from MedChemExpress (MCE). EasySepTM Mouse MDSC (CD11b^+^Gr-1^+^) isolation kit (19,867), EasySepTM Mouse CD45 Positive Selection kit (18,945) and EasyEightsTM EasySepTM Magnet (18,103) were purchased from STEMCELL Technologies. Ultrasonic cell disruptor was purchased from Xiaomei ultrasonic instrument (Kunshan) Co., Ltd. Live/Dead staining kit (Calcein acetoxymethyl ester and propidium iodide probes), 2′,7′-dichlorofluorescin diacetate (DCFH-DA) probes and DAPI (Beyotime, C1006) were purchased from solarbio. HE staining kit was purchased from AiBIxin Biotechnology Co., Ltd. (abs9217, China). Cell cycle staining kit (CCS012) were purchased from MultiScience Biotech Co., Ltd. 808 nm laser and 660 nm laser were purchased from BEIJING LASERWAVE OPTOELECTRONICS TECHNOLOGY.

### Cell lines and animals

SCC7 were purchased from UBIGENE Biosciences (Guangzhou, China) and genotype confirmed using STR sequence. Cells were cultured in Dulbecco’s Modified Eagle Medium (DMEM, Gibco), a 10% fetal bovine serum (FBS, Gibco), and 1% Penicillin and streptomycin (Gibco). According to the corresponding guidelines, a 5% CO_2_ concentration and a temperature of 37 ° C were the culture conditions to ensure cell survival. C3H/He mice (female, 6–8 week) were purchased from Experimental Animal Center of Southern Medical University. All procedures were approved and performed according to the guidelines of Institutional Animal Care and Use Committee of Southern Medical University.

### Myeloid derived suppressor cells (MDSCs) isolation and membranes harvest

Firstly, the femur was dissected from C3H tumor-bearing mouse. Cell suspension was harvested by irrigation and then filtered with 70 μm cell strainers (Becton & Dickinson) from bone marrow cavity. Next, EasySepTM Mouse MDSC (CD11b^+^Gr-1^+^) isolation kit and EasyEightsTM EasySepTM Magnet were used to separate CD11b^+^Gr-1^+^ MDSCs according to the instructions. The MDSCs membranes were harvested according to our previous work [30].

### Preparation of BDM

The bulk BP was dispersed in a NMP solution saturated with NaOH. Liquid-phase exfoliation was performed using an ultrasonic crusher in an ice bath. BP nanosheets, with a particle size of approximately 200 nm, were selected for subsequent experiments. Then, BP and Decitabine (BP: Decitabine = 1:5) were mixed in ultra-pure water without oxygen and stirred on a magnetic stirrer in the dark for 12 h. After undergoing centrifugation multiple times, the precipitate (BD, BP: Decitabine = 1:2.5) was redispersed in ultra-pure water without oxygen for further use. Finally, following the literature’s guidelines [31], the pre-prepared MDSCs cell membrane was coated onto the periphery of BD through ultrasound treatment according to the proportion (1 mg BD : 1 × 10^7^ membranes); subsequently, BDM was obtained by harvesting the precipitate through centrifugation at 15,000 rpm.

### Characterization of BDM

Transmission electron microscopy (TEM) images and element mapping were acquired by JEOL JEM2200FS (Japan). The dynamic light scattering (DLS), and Zeta potential experiments were detected by Zetasizer Nano-ZS (Malvern Instruments). The UV–vis absorption spectra was tested by UV–vis spectrophotometer (Agilent, Cary5000).

### Photothermal properties

To evaluate the photothermal properties of BDM, 500 µL ultra-pure water without oxygen containing different concentration BDM solution (0, 5, 10, 20, 40, 80 µg/mL based on BP) were irradiated under 808 nm laser (BEIJING LASERWAVE OPTOELECTRONICS TECHNOLOGY) with power density of 1.5 W/cm^2^ for 5 min. Then, the 500 µL ultra-pure water without oxygen containing 80 µg/mL BDM solution was irradiated under 808 nm laser with different power density (0.8, 1.0, 1.2, 1.5 W/cm^2^) for 5 min. The real-time temperature was monitored by infrared thermal imager (Fotric, Beijing, China). The photothermal stability of BDM were examined by six cycles (10 min per cycle) of laser irradiation “on” and “off” (80 µg/mL, 1.5 W/cm^2^).

### Photodynamic properties

To evaluate the photodynamic properties of BDM, 1,3-diphenylisobenzofuran (1, 3-DPBF) were used to detect the presence of singlet oxygen (one type of the reactive oxygen species). 500 µL ultra-pure water without oxygen containing 80 µg/mL BDM solution and 500 µL 1, 3-DPBF (50mM) were irradiated under 660 nm laser (BEIJING LASERWAVE OPTOELECTRONICS TECHNOLOGY) with power density of 150 mW for different time (0, 1, 2, 3, 4, 5 min). Then, 500 µL ultra-pure water without oxygen containing 80 µg/mL BDM solution and 500 µL 1, 3-DPBF (50mM) were irradiated under 660 nm laser with different power density (50, 100, 150 mW) for 5 min. After irradiation, the decrease in the absorption signal of 1,3-DPBF at λmax 420 nm were measured by UV–vis spectrophotometer (Agilent, Cary5000).

### Decitabine release of BDM

The dialysis method was employed to analyze the release of Decitabine in BDM with or without 808 nm laser (1.5 W/cm^2^) irradiation at pH 5.0. A total of 4mL of BDM solution (0.88 mg/mL) was evenly distributed into two separate dialysis bags, each containing a receiving medium volume of 100mL. Following laser irradiation for durations of 0, 2, 4, 8, and 12 h, 1mL samples were extracted from the receiving medium for analysis by UV-vis absorption spectrometer to measure the absorbance of released Decitabine. The content of released Decitabine was then calculated based on the standard curve.

### SDS-PAGE

In order to verify the surface proteins of MDSCs membrane, sodium dodecyl sulfate-polyacrylamide gel electrophoresis (SDS-PAGE) were performed. Firstly, the proteins of BD, MDSCs, MDSCs membrane, and BDM were extracted and measured with a bicinchoninic acid (BCA) kit (Beyotime). Then, all samples were heated at 95 °C for 5 min and 20 µg per sample was added into a 10% SDS-polyacrylamide gel, running at 80 V for 0.5 h and then 120 V for 1 h. Finally, before taking pictures, the gel was stained with Coomassie blue for 4 h and decolorated overnight by gentle shaking.

### Molecular dynamics simulation

Molecular dynamics simulations were performed by shiyanjia Lab (www.shiyanjia.com). They provide relevant experimental methods and data analysis strategies. The g_mmpbsa tool was used to calculate the binding energy [32, 33].

### CCK-8 assay

Firstly, SCC7 were seeded in 96-well plates with a density of 2 × 10^3^ cells per well and incubated for 24 h. Then, they were washed by PBS and incubated with different concentrations of decitabine or different materials (Con, BP, Decitabine, BD, BDM) with or without laser for 4 h (808 nm, 1.5 W/cm^2^, 5 min; 660 nm, 150mV, 5 min). Five replicate wells were designed for each group. Subsequently, the cells were continuously incubated for 20 h and then the cell viabilities were measured by CCK-8 kit according to manufacturer’s instructions. Maximum and minimum values were removed during data analysis.

### Immunofluorescence (IF) and immunohistochemistry (IHC)

Paraffin-embedded tissue samples underwent deparaffinization and antigen retrieval. The primary antibodies were PCNA (1:10000, CST, 13,110), HMGB1(CST, 6893 S), CRT (CST, 12,238 S), Caspase 3 (1:200, Proteintecch, 19677-1-AP). Live/Dead stain kit (Calcein acetoxymethyl ester and propidium iodide probes) and the probe 2′,7′-dichlorofluorescin diacetate (DCFH-DA) used to detect the ROS level were performed strictly following the instructions. IHC images were obtained with a digital pathology scanner (Leica), and IF images were taken with a confocal microscope (Leica).

### Cell cycle arrest analysis

The cells were seeded at a density of 5 × 10^5^ cells per well in 6-well plates, and four experimental groups were established: (1) Control (Con); (2) BP group; (3) Decitabine group; (4) BD group; and (5) BDM group. Each group was replicated three times. After incubation for 24 h, the cells were washed once with 1 mL of phosphate-buffered saline (PBS). Subsequently, they were treated with 1mL DNA staining solution and 10 µL permeabilization solution, blending vortex oscillation for 5–10 s. It could be detected after 30 min incubation at room temperature in the dark.

### In vivo imaging

Six female C3H mice (6–8 weeks) were used to establish tumor-bearing mouse model, SCC7 cells (1 × 10^7^/mL cells were suspended in serum-free cell medium) were injected subcutaneously into the flank of C3H mouse. A week later, the tumor volume reached ∼ 100 mm^3^, tumor-bearing C3H mice were randomly divided into two groups and received an intravenous (*i.v.*) injection of 100 µL PBS or PBS containing BDM (80 µg/mL) labeled by Cy5. The fluorescence images were acquired at different time points (1, 4, 8,12 and 24 h) by In Vivo FX PRO (Bruker, Germany). After 24 h, both groups of mice were euthanized, and their hearts, livers, spleens, lungs, kidneys, and tumors were harvested for in vivo imaging.

### In vivo biosafety

Female C3H mice (6–8 weeks, n = 3 per group) received an *i.v.* injection of 100 µL PBS, or PBS containing BDM (80 µg/mL) twice a week. Body weights were monitored every other day. 20 days later, all mice were euthanized and harvested their blood samples and major organs, including hearts, livers, spleens, lungs, kidneys and brains. Common blood routine and blood biochemical indicators were measured by using a blood biochemical autoanalyzer (7080, HITACHI, Japan). Organs were fixed in 4% neutral buffered formalin for hematoxylin and eosin (H&E) stain, and scanned by Aperio VERSA (Leica, Germany).

### In vivo treatment

C3H tumor-bearing mouse model were constructed following the above statement in “In vivo imaging” part. Thirty mice were randomly separated into six groups (n = 5 per group), including G1: Con, G2: Laser, G3: BD, G4: BDM, G5: BD + Laser, G6: BDM + Laser. After 4 h injection, the mice were treated with 808 nm laser (1.5 W/cm^2^) and 660 nm laser (150 mW) for 5 min in G2, 5 and 6. The real-time temperature in tumor was recorded by infrared thermal imager (Fotric, Beijing, China). The body weights and tumor volume of mice were measured every other day. All mice were treated twice a week for 2 weeks. After that, the mice were euthanized and the tumors were removed. After their pictures were took, tumor specimens of G1 and G6 were divided into 4 parts: (1) Fixed in 4% neutral buffered formalin for IHC analysis. (2) Partial tumor tissues were kept on ice until use for subsequent flow cytometry analysis. (3) A quarter of the tumors were frozen for further experiments. (4) The remaining tumors were snap-frozen in liquid nitrogen, and three samples from each group were randomly selected for RNA sequencing analysis. While the tumors of G2-5 just need to separate for three parts: part 1), 2) and 3).

### RNA sequencing

Total RNA of tumor tissues in G1 and G6 was extracted by NovoTech. Followed by quality-checked for total RNA and libraries were sequenced on an Illumina Novaseq platform. Subsequently, relevant data analysis and packaging of the analysis results files.

### Flow cytometry

For flow cytometric analysis, single cell suspensions of tumors were first prepared according to our previous work [29]. Subsequently, EasySepTM Mouse CD45 Positive Selection kit (18,945) and EasyEightsTM EasySepTM Magnet (18,103) were used to enrich CD45^+^ immune cells infiltrated in TME. After that, the cells were divided into two panels. Panel 1 was stained with following antibodies: Live/Dead (ECD, Biolegend), CD45 (APC-Cy7; clone 30-F11), CD11b (APC; clone M1/70), Gr-1 (BV650; clone RB6-8C5), F4/80 (BV421; clone BM8), CD86 (PE; clone GL1) (all from Becton & Dickinson), CD206 (BV785; clone BM8), CD11c (PE-CY7; clone B-ly6), CD103 (PE; clone M290); Panel 2 was stained with following antibodies: Live/Dead (ECD, Biolegend), CD45 (APC-Cy7; clone 30-F11), CD8a (BB515; clone 53–6.7), CD4 (BB700; clone RM4-5), CD279 (PE-Cy7; clone RMP1-30). The frequency or MFI of immune cells were measured by a CytoFLEX flow cytometer (Beckman). The results were analyzed by FlowJo (Tree Star).

### Statistical analysis

Data analyses were performed using Graph Pad Prism version 9.0 for Windows (Graph Pad Software Inc, La Jolla, CA) and Origin 2021for Windows. Normality and Lognormality tests, Unpaired t-test, one-way ANOVA with Dunnett’s or Tukey’s multiple comparison tests and two-way ANOVA with Dunnett’s or Sidak’s multiple comparisons test was used to analyze significant differences. Dates were represented as the mean ± SD. Differences (*p* < 0.05) were considered statistically significant. (*, *p* < 0.05; **, *p* < 0.01; ***, *p* < 0.001; ****, *p* < 0.0001; ns, not significant).

## Results and discussion

### Preparation and characterization of BDM

**Fig. 1:**
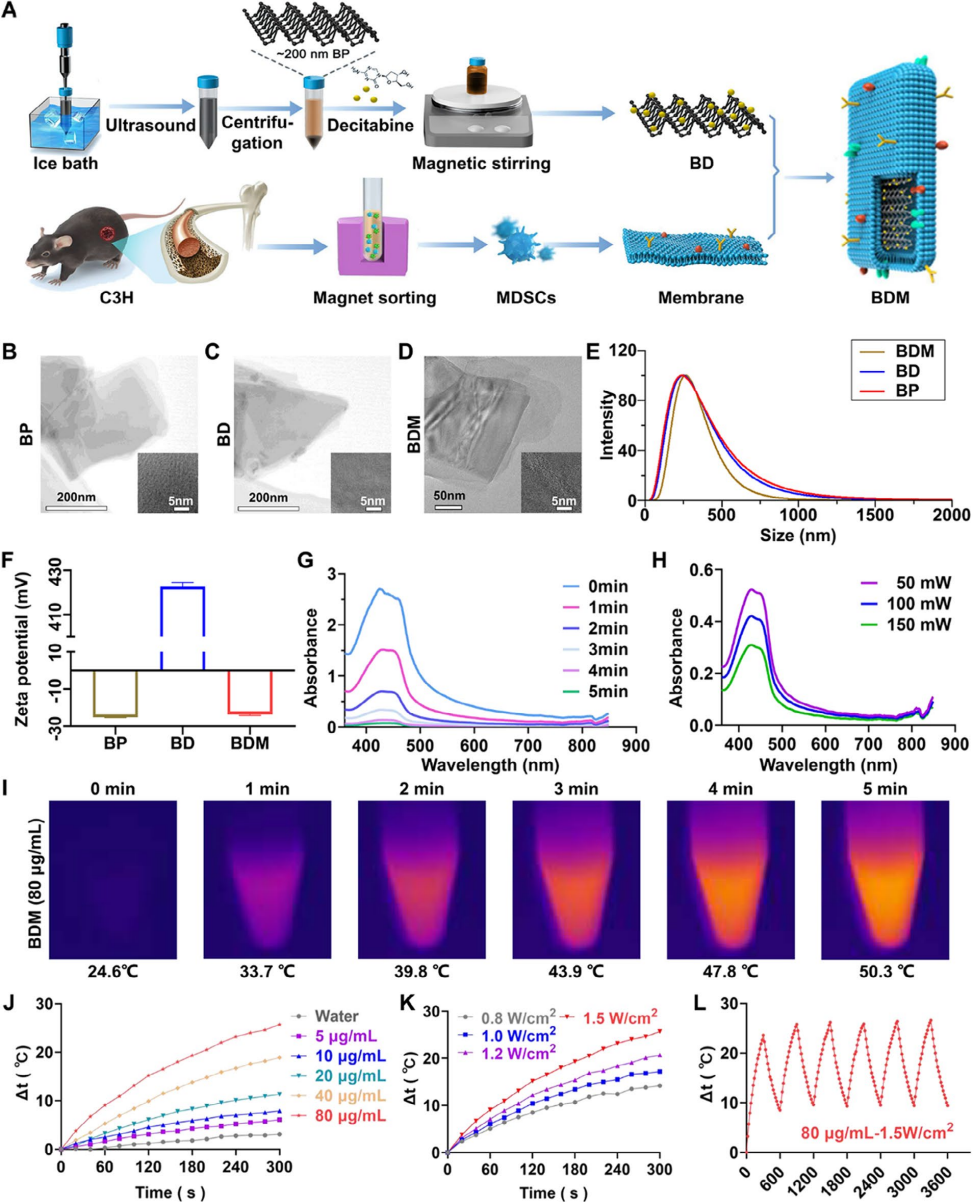
Preparation and Characterization of BDM. (**A**) Schematic diagram of the fabrication of BDM nanosheets. (**B-D**) TEM images of BP, BD and BDM. (**E**) Hydrodynamic diameter of BP, BD and BDM measured by dynamic light scattering. (**F**) Zeta potentials of BP, BD and BDM. (**G**) Absorption of DPBF in the presence of BDM (80 µg/mL) under irradiation (660 nm, 150mW) with different time. (**H**) Absorption of DPBF in the presence of BDM (80 µg/mL) under irradiation (660 nm, 5 min) with different power. (I) Photothermal images of BDM (80 µg/mL) in different times with laser irradiation (808 nm, 1.5 W/cm^2^). (**J-K**) Heating curves of BDM under different Concentrations and intensities. (**L**) Photothermal stability of BDM. All experiments were independently repeated in triplicate

As shown in Fig. 1A, the preparation of BDM can be briefly divided into three steps: (1) ∼ 200 nm BP, obtained by liquid phase exfoliation [34], were successfully loaded with decitabine (BD). (2) MDSCs were isolated from the bone marrow of tumor-bearing mice by magnetic sorting, and then the plasma membrane of MDSCs was prepared by hypotonic lysis and differential centrifugation. (3) BD nanoparticles were encapsulated with MDSCs membrane by ultrasound, and the uncoated membranes were removed by centrifugation at 15,000 rpm.

To confirm the successfully fabrication of BDM, the morphologies of BP, BD and BDM were characterized by transmission electron microscopy (TEM) (Fig. 1B-D). As Shown in Fig. 1D, BD was surrounded by a faint shadow, indicating the well-encapsulation of MDSCs membranes. The dynamic light scattering (DLS) was performed to detect the hydrodynamic diameter and zeta potentials of different nanoparticles. The mean diameters of BP, BD and BDM were ∼ 238 nm, ∼ 245 nm and ∼ 264 nm (Fig. 1E). The zeta potentials were − 25.22, 422.7 and − 23.58 mV, respectively (Fig. 1F). Sodium dodecyl sulfate-polyacrylamide gel electrophoresis (SDS-PAGE) stripes further confirmed that the whole synthesis process did not affect the expression of MDSCs membrane proteins (Figure S1). Due to its great photothermal conversion efficiency and singlet oxygen generation efficiency properties, BP has been developed as a promising nanomaterial for cancer treatment under 660 and 808 nm laser irradiation [35]. 80 µg/mL BDM was irradiated with 660 nm laser for several minutes at different powers, and the generation of effective reactive oxygen species (ROS) was detected by 1,3-diphenylisobenzofuran (DPBF) (Fig. 1G, H). The results showed that 150 mW, 5 min is capable to produce large amounts of ROS. By monitoring the stability of BP, BD and BDM at 0, 1, 3, 5, 10 days, we found that all of them had a slow degradation rate (Figure S2). As for the photothermal performance, the heating curves exhibited a concentration and power density dependence as the concentration of BDM increased from 0 to 80 µg/mL and the 808 nm laser power changed from 0.8 to 1.5 W/cm^2^ (Fig. 1I, J). In addition, BDM displayed great photothermal stability through six ON/OFF cycles (Fig. 1K). As Shown in Fig. 1L, Figures S3 and S4, when 80 µg/mL BP, BD and BDM were irradiated under 1.5 W/cm^2^ 808 nm laser for 5 min, the temperature increased by 54.5℃, 52.8℃ and 50.3℃ within 5 min respectively. It is sufficient to eradicate the tumor and avoid damage to the peritumoral normal tissue [36, 37]. Additionally, analysis using a dialysis bag demonstrated that laser irradiation effectively enhanced the release of Decitabine from BDM (Figure S5).

**Fig. 2:**
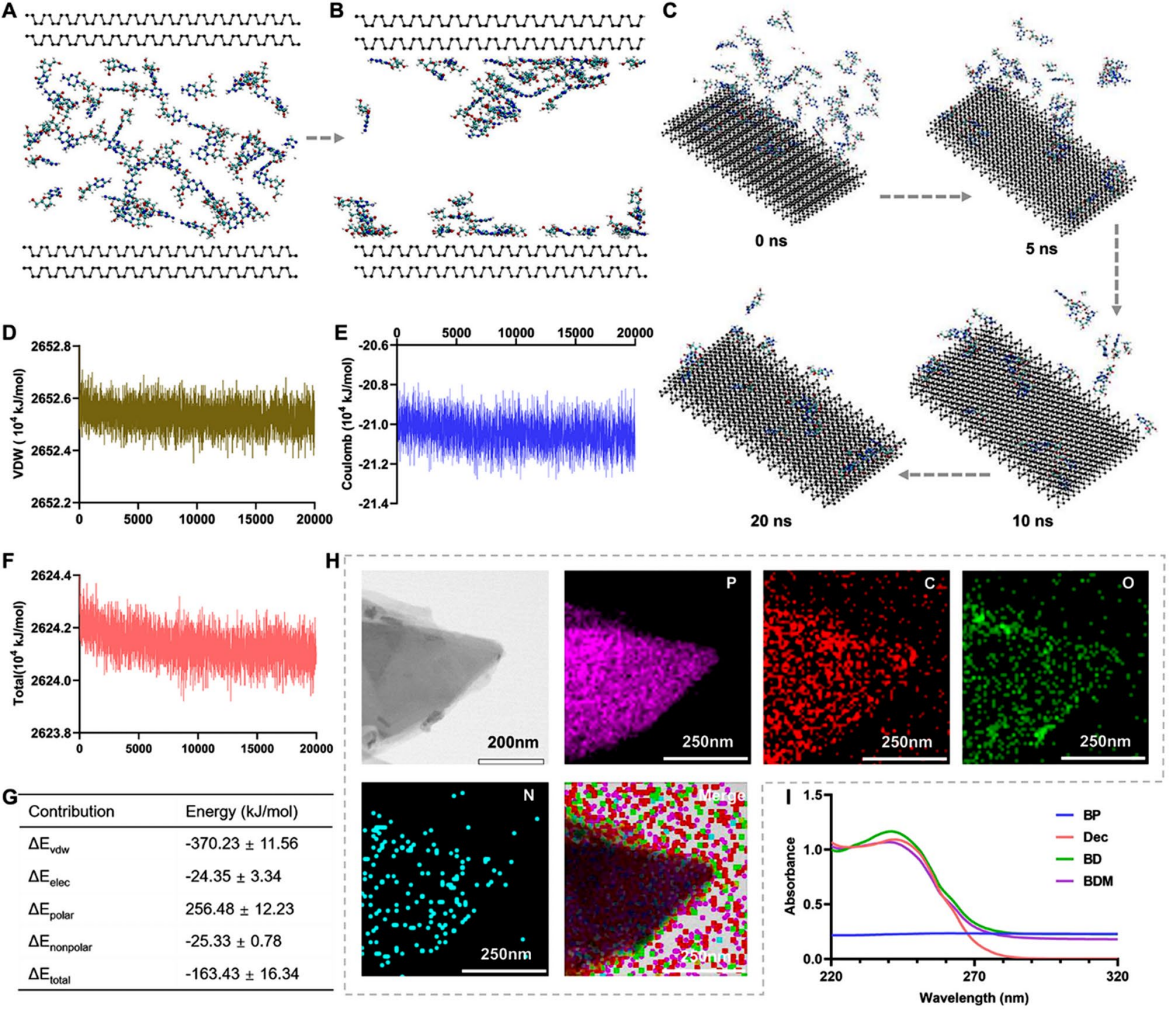
The drug loading capacity of BP. (**A-B**) Schematic images of BP before and after decitabine was loaded. (**C**) The interaction of BP and decitabine within 0, 5, 10 and 20ns. (**D-F**) The van der Waal force, column force and total force that contributed to the interaction between BP and decitabine. (**G)** The energy changes between BP and decitabine. (**H**) TEM mapping of BD. Scale bar = 200 and 250 nm. (**I**) UV–vis–NIR spectra of BP, decitabine (Dec), BD and BDM.

Due to the large surface and specific surface ratio of 2D nanomaterials, black phosphorus is considered as a promising nanoplatform for drug delivery [38, 39]. To verify the feasibility of loading decitabine on black phosphorus, molecular dynamic simulation was performed to predict their mutual binding interactions [40]. The results showed that within 20ns, most of the drug molecules of decitabine were successfully adsorbed on the surface of black phosphorus (Fig. 2A-C). The interaction of BP with decitabine was mainly attributed to van der Waal forces (Fig. 2D-G). TEM mapping was performed to examine the elements of BP and BD, which revealed significant N distributed on BP (Fig. 2H, S6, S7). The UV-Vis spectra further confirmed that when BP was loaded with decitabine, an obvious absorption peak of decitabine exhibited at 242 nm no matter whether it was coated with MDSCs cell membrane or not (Fig. 2I).

### In vitro anti-tumor effect analysis

**Fig. 3:**
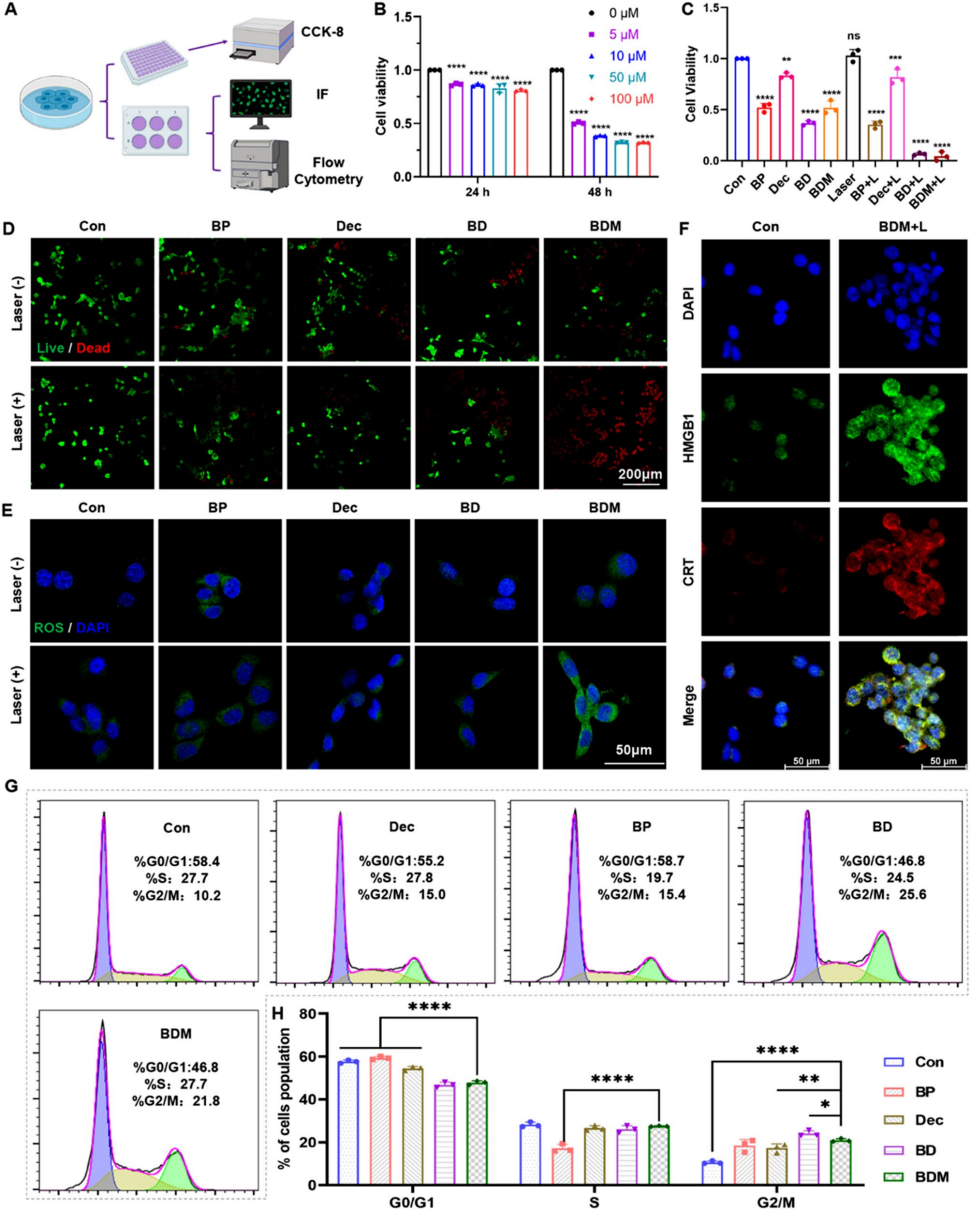
In vitro anti-tumor effect analysis of BDM. (**A**) Schematic diagram of the in vitro anti-tumor assay of BDM. (**B**) The cell viability of SCC7 stimulated with decitabine under different concentrations following the instruction (n = 3 per group, two-way ANOVA with Dunnett’s multiple comparisons test was used). (**C**) The cell viability of SCC7 under different treatments (n = 3 per group, one-way ANOVA with Dunnett’s multiple comparisons test was used). (**D**) Immunofluorescence images of live/dead staining of SCC7 cells with or without 808 nm (1.5 W/cm^2^, 5 min) and 660 nm (150mW, 5 min) laser irradiation treatment. Scale bar = 100 μm. (**E**) Immunofluorescence images of ROS staining of SCC7 cells with or without 660 nm (150mW, 5 min) laser irradiation treatment. Scale bar = 50 μm. (**F**) Immunofluorescence images of HMGB1 and CRT expression on SCC7 cells with BDM nanoparticles (80 µg/mL) co-incubation under 808 nm (1.5 W/cm2, 5 min) and 660 nm (150mW, 5 min) laser irradiation treatment. Scale bar = 50 μm. (**G-H**) Cell cycle related flow cytometry analysis of SCC7 treated for 24 h (G1: Con, G2: BP, G3: BD, G4: BDM). The distribution of cells in G1, S, G2 phase (n = 3 per group, two-way ANOVA with Dunnett’s multiple comparisons test was used). All experiments were independently repeated in triplicate. (**, *p* < 0.01; ***, *p* < 0.001; ****, *p* < 0.0001; ns, not significant)

The methods depicted in Fig. 3A were performed to validate the anti-tumor effect in vitro. As indicated, decitabine cytotoxicity was assessed on SCC7 cells for 24 and 48 h (Fig. 3B). Subsequently, the CCK-8 assay results convincingly demonstrated that the combination of BP-based nanomedicine with 808 nm + 660 nm laser treatment significantly enhanced the anti-tumor effect (Fig. 3C). Immunofluorescence images of live/dead staining were utilized to elucidate the tumor cell killing capability. Notably, when receiving laser irradiation, the difference observed between the control and decitabine groups was negligible. In contrast, an increase in dead cells was observed in the BP, BD, and BDM groups after 5 min of 808 nm and 660 nm laser irradiation, respectively (Fig. 3D). The probe known as “2’,7’-dichlorofluorescin diacetate” (DCFH-DA) was employed to detect ROS generation after incubation with various components of BDM with or without exposure to a 660 nm laser. Remarkably, BDM + Laser group exhibited a significant promotion in ROS production (Fig. 3E). Studies have demonstrated that PTT and PDT can induce immunogenic cell death (ICD) and trigger the release of damage-associated molecular pattern molecules, such as high mobility group B1 (HMGB1) and calreticulin (CRT), thereby enhancing anti-tumor immunity [41]. Significant release of HMGB1 and CRT was detected by confocal fluorescence microscopy, as shown in Fig. 3F, indicating the potential of BDM + laser to induce ICD. Given that BP and decitabine have been reported to induce tumor cell apoptosis through G2/M cell cycle arrest [42–44], we investigated whether BP and decitabine exhibit synergistic anti-tumor effects by flow cytometry to verify changes in cell proportions during each phase of the cell cycle. The results revealed that the proportion of G2/M cells was significantly higher in the BP, BD, and BDM groups compared with the control group. However, the presence of cell membrane appeared to slightly attenuate the cell cycle arrest effect of BDM relative to what was observed in the BD group (Fig. 3G, H).

### Tumor-targeting ability of BDM

**Fig. 4:**
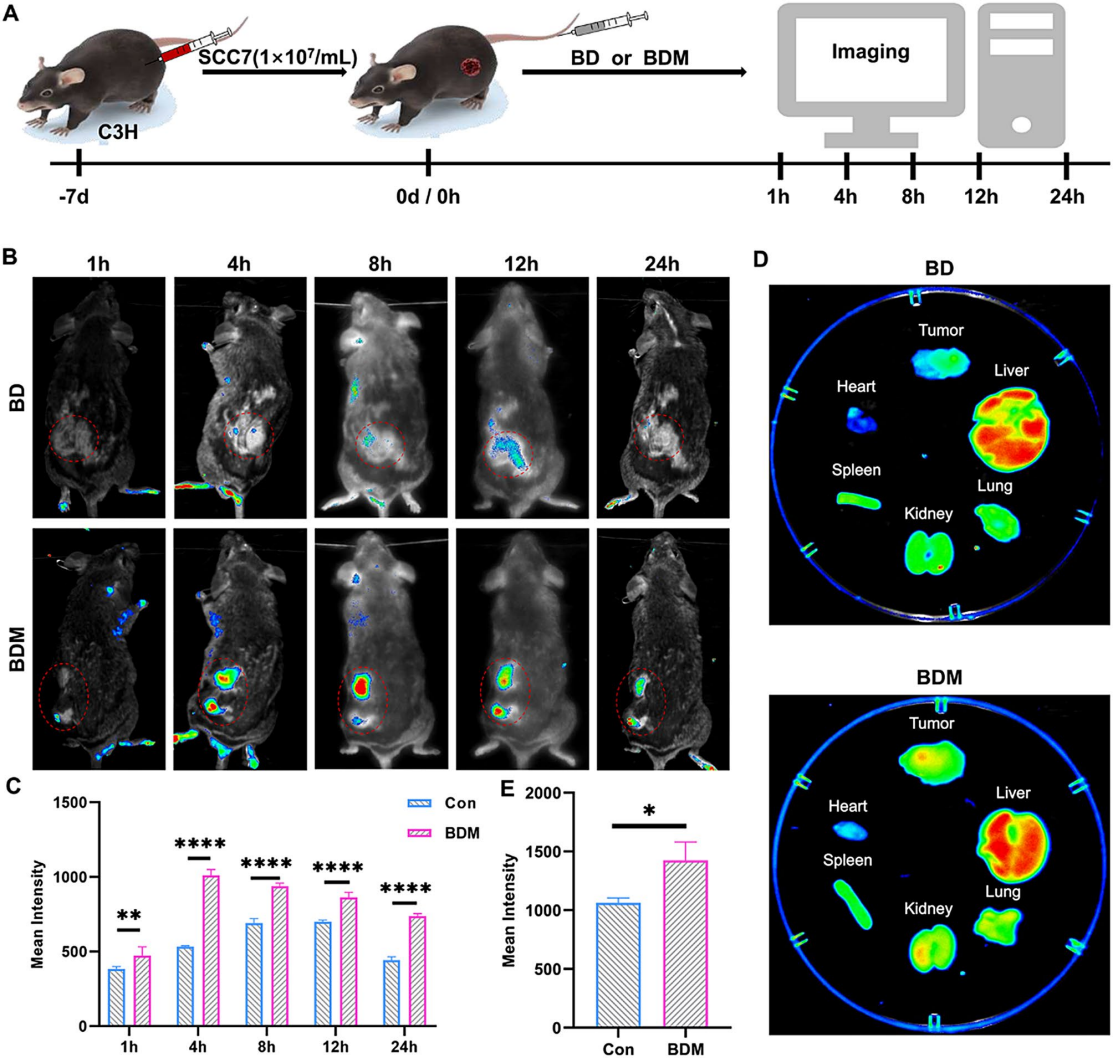
The active tumor-targeting ability of BDM. (**A**) Schematic diagram of in vivo imaging experiment: Tumor-bearing C3H mice were treated with BD and BDM label with Cy5 (*i.v.*, 80 µg/mL), and then the fluorescence images were taken at 1, 4, 8, 12 and 24 h after injection. (**B**) In vivo fluorescence images of SCC7 tumor-bearing C3H mice taken at different times. (**C**) Quantitative mean fluorescence intensities of tumors in different groups at various time points. (**D**) Represent ex vivo fluorescence images of tumors and main organs dissected from different group at 24 h post-injection. (**E**) Quantitative mean fluorescence intensities of ex vivo tumors in different groups at 24 h post-injection. (*, *p* < 0.05; **, *p* < 0.01; ***, *p* < 0.001, ns, not significant)

The active tumor-targeting ability of BDM has been attributed to the gene expression of chemokines and cytokine receptor proteins on the surface membrane of MDSCs. Therefore, BDM accumulates in tumor tissue through blood circulation, responding to cytokines and chemokines produced by tumor cells and surrounding normal tissue cells. To further validate the active targeting ability of BDM, we injected 80ug/mL BD and BDM (labeled with Cy5) into C3H tumor bearing mice through the tail vein and performed in vivo imaging at 0, 1, 4, 8,12, and 24 h after injection (Fig. 4A). The fluorescence intensity of Cy5 accumulated in tumor tissue peaked after 4 h (Fig. 4B, C). Mice were euthanized 24 h after injection, and tumors and major organs were removed for in vivo imaging analysis, quantitative results showed that BDM significantly enriched in tumor tissue compared with BD (Fig. 4D, E).

### Assessment of the in vivo antitumor efficacy of BDM

**Fig. 5:**
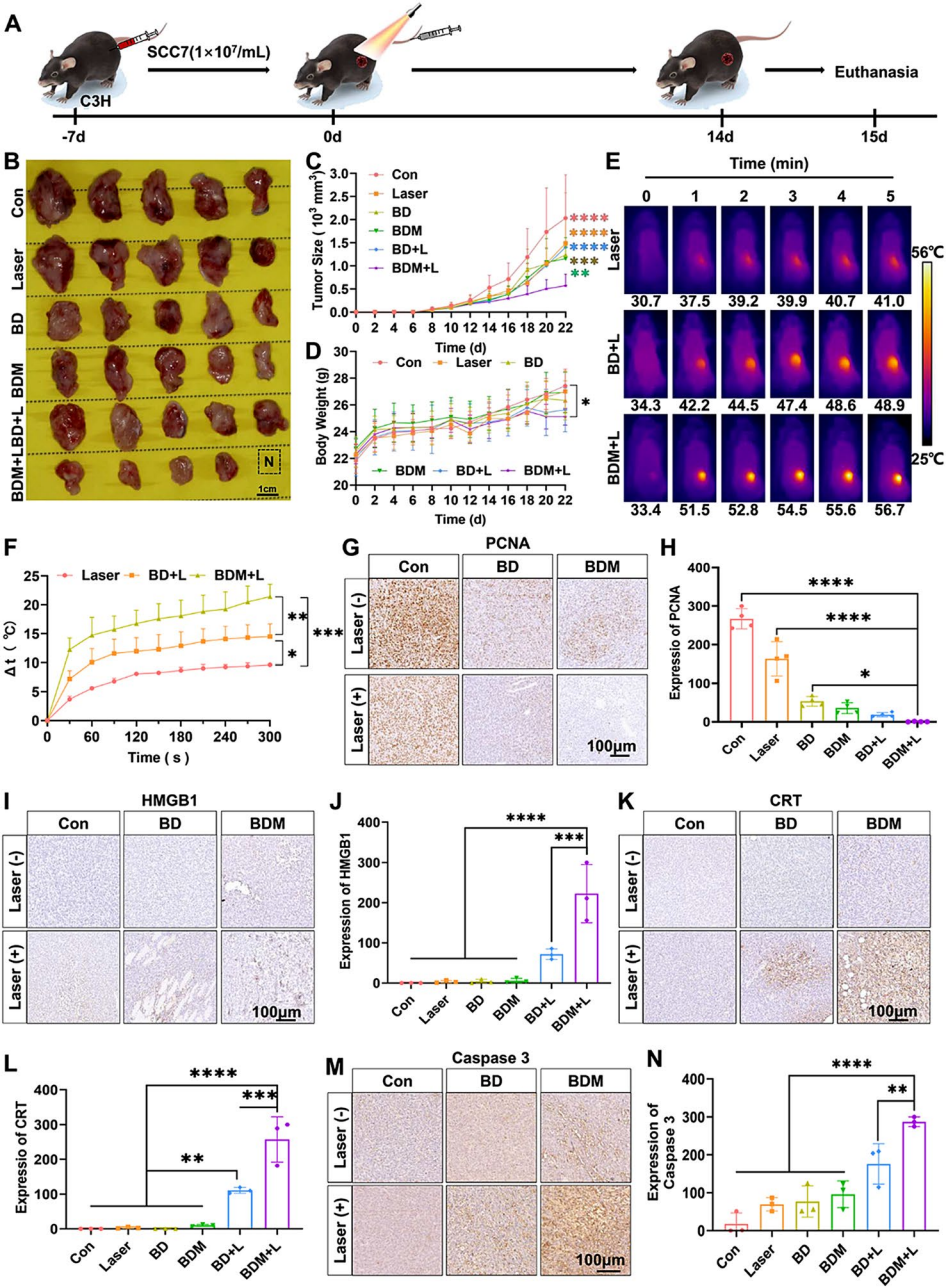
Assessment of the in vivo antitumor efficacy of BDM. (**A**) Schematic diagram of in vivo treatment: Tumor-bearing C3H mice were treated for four times within 14 days. After that, mice were euthanasia at 15 days. (G1: Con, G2: Laser (L), G3: BD, G4: BDM, G5: BD + L, G6: BDM + L) (**B**) Images of tumors dissected from different groups. (**C**) The tumor size in different treatment group (n = 4 per group, two-way ANOVA with Dunnett’s multiple comparisons test was used). (**D**) The change of body weight in different treatment group (n = 4 per group, two-way ANOVA with Dunnett’s multiple comparisons test was used). (**E**) Representative thermographic images in G2, 5, 6 under 808 nm (1.5 W/cm^2^, 5 min) and 660 nm (150mW, 5 min) laser irradiation treatment at 4 h post *i.v.* injection of PBS, BD and BDM. (**F**) The change of temperature (Δt) in G2, 5, 6 (n = 5 per group, two-way ANOVA with Dunnett’s multiple comparisons test was used). (**G**) The representative immunohistochemical staining images and quantitative analysis of PCNA expressed in tumors in G1-6. Scale bar = 100 μm. (**H**) The IHC analysis of PCNA expression per µm^2^ in G1-6 (n = 3 per group, one-way ANOVA with Dunnett’s multiple comparisons test was used). (**I**) The representative immunohistochemical staining images and quantitative analysis of HMGB1 expressed in tumors in G1-6. Scale bar = 100 μm. (**J**) The IHC analysis of HMGB1 expression per µm^2^ in G1-6 (n = 3 per group, one-way ANOVA with Dunnett’s multiple comparisons test was used). (**K**) The representative immunohistochemical staining images and quantitative analysis of CRT expressed in tumors in G1-6. Scale bar: 100 μm. (**L**) The IHC analysis of CRT expression per µm^2^ in G1-6 (n = 3 per group, one-way ANOVA with Dunnett’s multiple comparisons test was used). (**M**) The representative immunohistochemical staining images and quantitative analysis of Caspase 3 expressed in tumors in G1-6. Scale bar = 100 μm. (**N**) The IHC analysis of Caspase 3 expression per µm^2^ in G1-6 (n = 3 per group, one-way ANOVA with Dunnett’s multiple comparisons test was used). (*, *p* < 0.05; **, *p* < 0.01; ***, *p* < 0.001; ****, *p* < 0.0001)

BDM-mediated anti-tumor efficiency was assessed in C3H tumor bearing mice. According to the flow chart (Fig. 5A), mice were randomly divided into 6 groups (5 in each group): G1: PBS; G2: laser (L, 660 nm, 150 mW, 5 min; 808 nm, 1.5 W/cm^2^, 5 min); G3: BD; G4: BDM; G5: BD + L; G6: BDM + L, and they were treated with 80ug/mL BD and BDM twice a week for 2 weeks. According to the results of in vivo imaging, laser irradiation was conducted 4 h after the intravenous injection. After four treatments, the mice were euthanized and tumor tissues were harvested for subsequent analysis. Consistent with the dissected tumor tissue shown in Fig. 5B, tumor growth was significantly inhibited in the BDM + L group (Fig. 5C), whereas body weight changes were negligible in each group (Fig. 5D). Meanwhile, representative thermograms showed that the temperature in the BDM + L group was 56.7 ° C, which was high enough to cause a significant PTT effect (Fig. 5E, F). It also reflects the active targeting ability of BDM, which is consistent with the results of in vivo imaging. The results of subsequent biosafety experiments showed that there were no significant changes in body weight, blood routine and blood biochemical indicators in the BDM group, and no significant histological changes were observed in the results of HE staining of the heart, liver, spleen, lung, kidney and brain, indicating a low risk of side effects (Figure S8-S11). Immunohistochemical (IHC) staining showed that the expression of PCNA, an indicator related to tumor cell proliferation, was significantly decreased after treatment (Fig. 5G, H). PTT-mediated ICD significantly induce the release of HMGB1 and CRT in a damage-related molecular pattern to promote anti-tumor immunity. Decitabine and BP can also induce tumor cell apoptosis [45, 46]. The results showed that the expressions of HMGB1 and CRT were significantly increased in the BDM + L group (Fig. 5I-L), and the expression of caspase 3, which is closely related to the occurrence of apoptosis [47], was also significantly increased (Fig. 5M, N). Thus, the therapeutic strategy of BDM + L can enhance the immunogenic cell death and apoptosis of tumor cells.

**Fig. 6:**
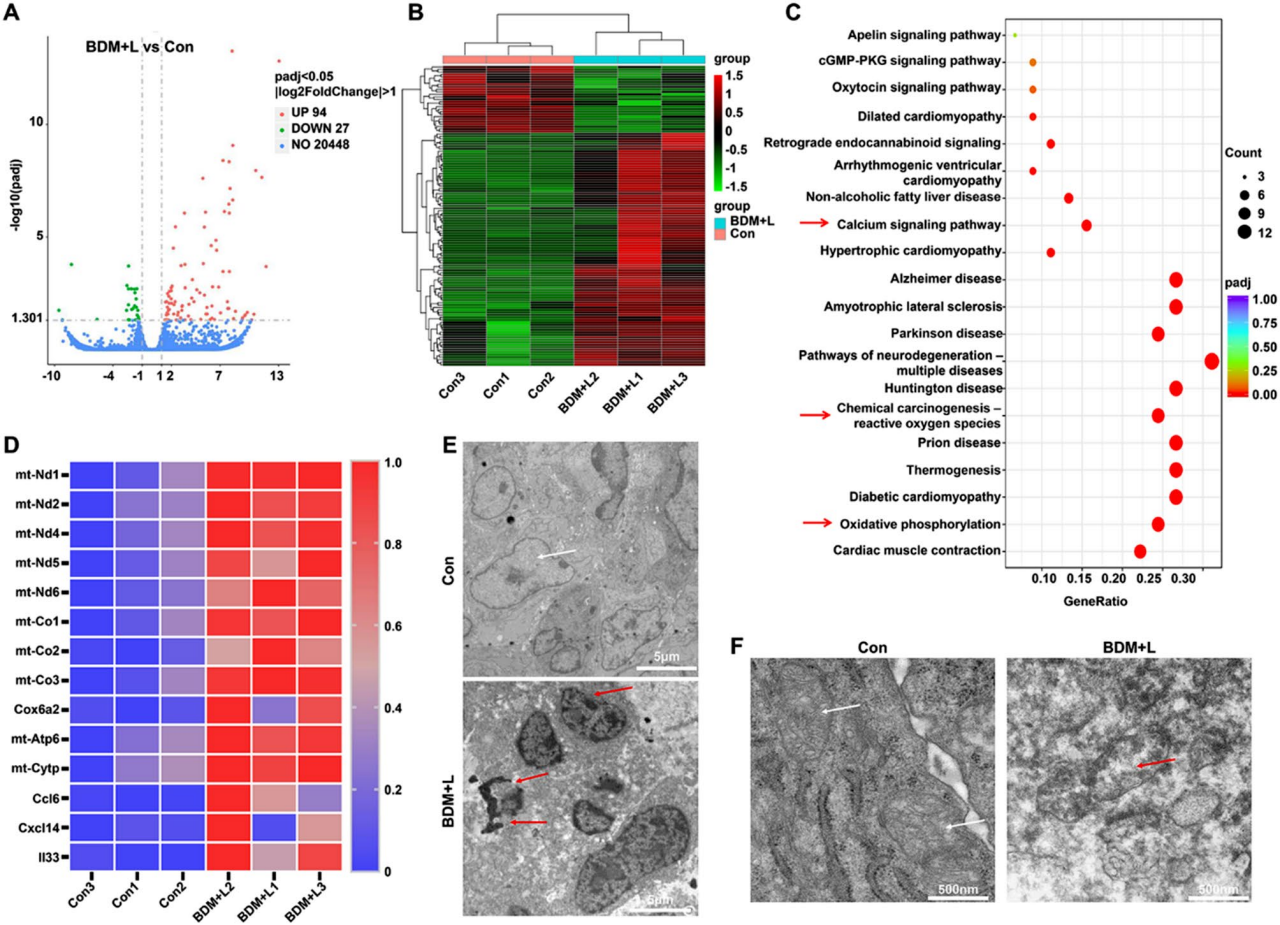
RNA-Sequence of tumor tissues revealed the mechanism of BDM enhanced anti-tumor effect. (**A**) The volcan map of con and BDM + L group (n = 3). (**B**) The heatmap of significant genes changed after BDM + L treatment (n = 3, *p* < 0.05). (**C**) The KEGG analysis in con and BDM + L group (Red arrows mean signaling pathways that we interested). (**D**) The heatmap of selected genes related to above associated signaling pathway (n = 3). (**E**) Bio-TEM images of tumor cells in con and BDM + L group. The white arrows point to normal nuclei, the red arrows point to the nuclei with damaged nuclear membranes. Scale bar = 5 μm. (**F**) Bio-TEM images of mitochondria in con and BDM + L group (n = 3). The white arrows point to the normal mitochondria, the red arrows point to the damaged mitochondria, characterized by the disappearance of the mitochondrial ridge structure and evident cavitation. Scale bar = 500 nm

The subsequent RNA sequencing results of tumor tissue further revealed that, compared with the control group, 94 genes were up-regulated and 27 genes were down-regulated after BDM + L treatment (Fig. 6A, B). KEGG enrichment analysis showed that after BDM + L treatment, those genes can be significantly enriched in Calcium signaling pathway, Chemical carcinogenesis-reactive oxygen species and oxidative phosphorylation related signaling pathways were significantly up-regulated (Fig. 6C). Personalized analysis of genes related to the above-mentioned signaling pathways showed that mitochondrial-related genes changed significantly (Fig. 6D). Therefore, it can be speculated that the upregulation of ROS expression leads to mitochondrial oxidative stress, which leads to mitochondrial damage and cell death. Subsequent results of biological transmission electron microscopy (Bio-TEM) of mitochondria and tumor cells also confirmed our conjecture. Electron microscopy showed that BDM + L treatment resulted in damage to the nuclear membrane of tumor cells (Fig. 6E). And mitochondrial morphology was significantly altered in the BDM + L group, characterized by loss of mitochondrial ridge structure, decreased substrate electron density, and evident cavitation (Fig. 6F).

### BDM-mediated antitumor immunity

**Fig. 7:**
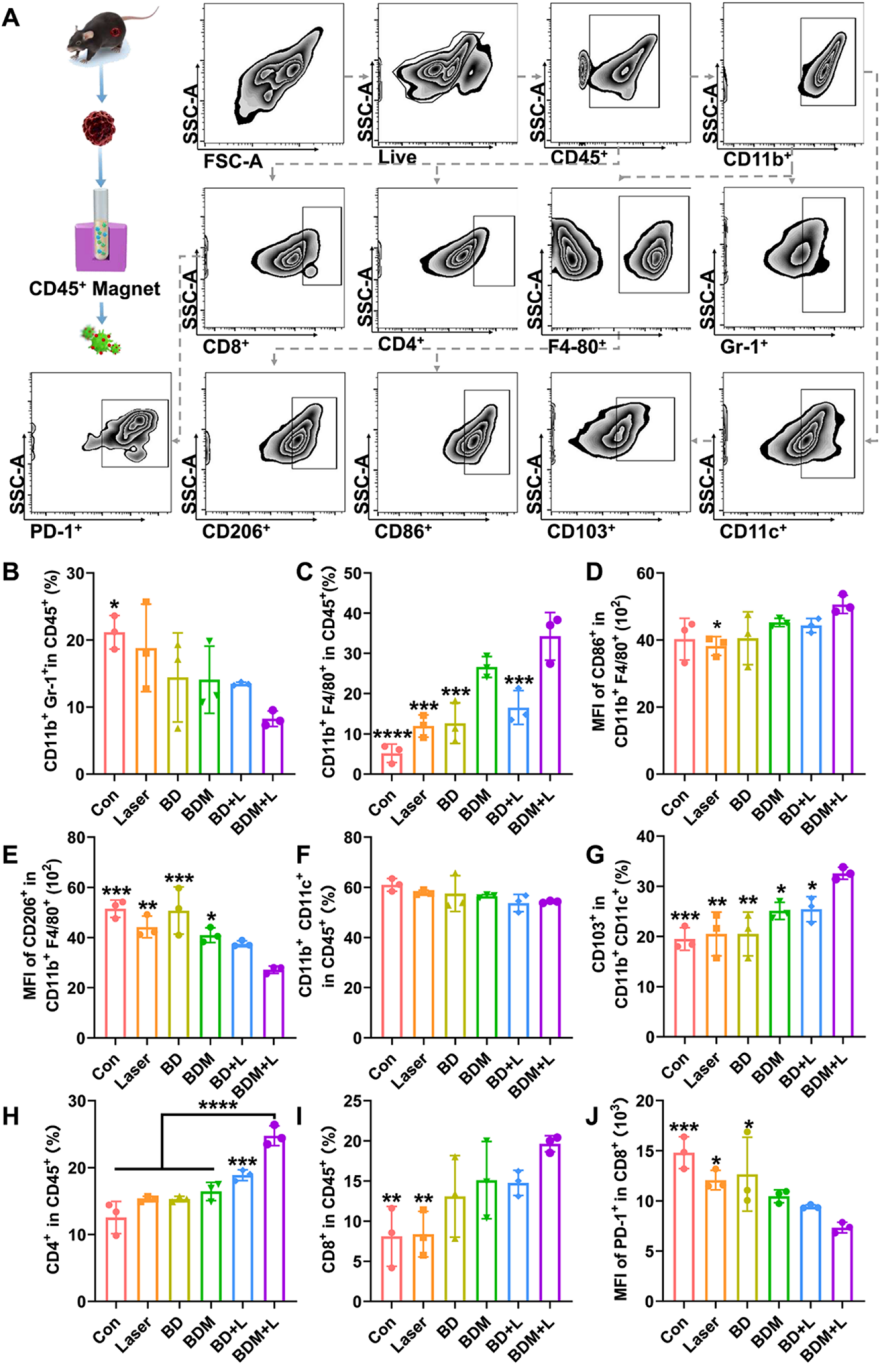
The infiltration of different immune cell subsets in tumor microenvironment of SCC7 tumor-bearing C3H mice. (G1: Con, G2: Laser (L), G3: BD, G4: BDM, G5: BD + L, G6: BDM + L) (**A**) The gating strategy for different immune cell subsets in tumor microenvironment of SCC7 tumor-bearing C3H mice (n = 3 per group). The percentage of tumor-infiltrating (**B**) CD11b^+^ Gr-1^+^ MDSCs, (**C**) CD11b^+^ F4/80^+^ Macrophages. The MFI of tumor-infiltrating (**D**) CD86^+^ CD11b^+^ F4/80^+^ M1-like Macrophages and (**E**) CD206^+^ CD11b^+^ F4/80^+^ M2-like Macrophages, the percentage of tumor-infiltrating (**F**) CD11b^+^ CD11c^+^ DCs, (**G**) CD103^+^ CD11b^+^ CD11c^+^ Matured DCs, (**H**) CD45^+^ CD4^+^ T cells and (**I**) CD45^+^ CD8^+^ T cells were compared. The MFI of tumor-infiltrating (**J**) CD45^+^ CD8^+^ PD-1^+^ T cells. (n = 3 per group, one-way ANOVA with Dunnett’s multiple comparisons test was used. *, *p* < 0.05; **, *p* < 0.01; ***, *p* < 0.001; ****, *p* < 0.0001)

The presence of an immunosuppressive TME and inadequate infiltration of cytotoxic T lymphocytes are the primary obstacles in effective tumor treatment [41]. Phototherapy mediated by BP nanomaterials facilitates the establishment of a pro-immune microenvironment [48]. Our experimental findings also demonstrate that BDM + L treatment enhances ICD-induced anti-tumor immune responses. Previous studies have reported that BP nanomaterials can modulate NK cells, DC cells, CD4^+^ T cells, and CD8^+^ T cells to elicit an anti-tumor immune response [49, 50]. Furthermore, the BP nanosystem not only can induce DC maturation and promotes T cell infiltration through ICD [20, 51], but also can promote the transformation of M2-like macrophages into M1-like macrophages with pro-inflammatory and anti-tumor phenotype to alleviate the immunosuppressive tumor microenvironment [52, 53]. Additionally, the secretion of proinflammatory cytokines IL-12 and TNF-α by M1 macrophages further enhances DC maturation [20].Therefore, flow cytometry was performed to monitor changes in various immune cell populations within the TME. The gating strategy for flow cytometry is shown in Fig. 7A. Subsequent analysis based on this strategy revealed a significant reduction in the proportion of CD11b^+^ Gr-1^+^ MDSCs after BDM + L treatment (Fig. 7B, S12), which may be attributed to the occupying effect of BDM. Although the proportion of total CD11b^+^ F4/80^+^ macrophages increased (Fig. 7C, S13), no significant change was observed in CD86^+^ M1 macrophages (Fig. 7D, S14). Conversely, a decreased proportion of tumor-promoting CD206^+^ M2 macrophages was observed (Fig. 7E, S15). The proportion of CD11b^+^ CD11c^+^ DCs did not change significantly (Fig. 7F, S16). However, a significant increase was observed in CD103^+^ CD11b^+^ CD11c^+^ DCs (Fig. 7G, S17). Studies have shown that HMGB1 and CRT can promote the conversion of immature DC into anti-tumor CD103^+^ DCs [54]. Subsequently, matured CD103^+^ DCs recognized T cells and increased the ratio of effector CD4^+^ T cells to exhausted CD8^+^ T cells (Fig. 7H, I, S18, S19). In addition, the expression of cell death protein 1 (PD-1), an exhaustion-related inhibitory receptor, was significantly decreased in the BDM + L group compared with the control group (Fig. 7J, S20).

## Conclusion

This study has successfully developed a BP-based nanoplatform encapsulated in the membrane of MDSCs (BDM) for drug delivery. The MDSCs membrane inherits the characteristics of MDSCs to evade host pathogen clearance and actively target the tumor microenvironment, enabling BDM to effectively target tumors. First of all, it achieves tumor hypothermia and hyperthermia through PTT. Secondly, it induces the production of ROS through PDT, leading to mitochondrial damage and promoting tumor cell apoptosis. Furthermore, decitabine-mediated CDT induced tumor G2/M cell cycle arrest and enhances tumor cell apoptosis. Finally, BDM-mediated ICD promotes host anti-tumor immunity and further augments the anti-tumor therapeutic effect. At the same time, BDM had good biosafety and biocompatibility. Taken together, we constructed a safe and efficient nanoplatform BDM and realized multifunctional anti-tumor therapy. In the future, we believe that BDM nanoparticles will serve as a valuable option for clinical cancer treatment.

### Electronic supplementary material

Below is the link to the electronic supplementary material.

Supplementary Material 1

## Data Availability

No datasets were generated or analysed during the current study.
