# Supplementary material for: Biomimetic MDSCs membrane coated black phosphorus nanosheets system for photothermal therapy/photodynamic therapy synergized chemotherapy of cancer
Source: J Nanobiotechnology. 2024 Apr 12;22:174. doi: 10.1186/s12951-024-02417-4 (PMC11015563; doi:10.1186/s12951-024-02417-4)
Supplement: Supplementary file 1 — Supplementary Material 1 [file 12951_2024_2417_MOESM1_ESM.docx]

**Biomimetic MDSCs Membrane Coated Black Phosphorus Nanosheets System for Photothermal Therapy/Photodynamic Therapy Synergized Chemotherapy of Cancer**

**Supplementary Figures**

**Figure S1**

**
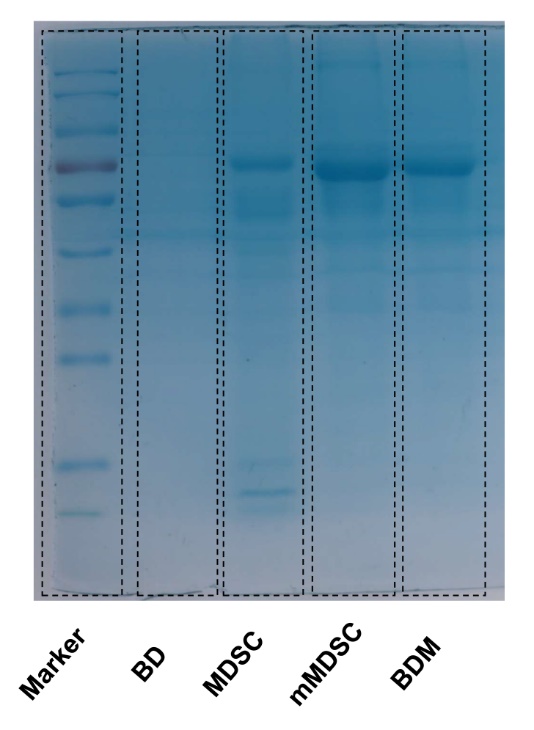
**

**Figure S1.** SDS-PAGE analysis of BD, MDSC, mMDSC and BDM.

**Figure S2**

**
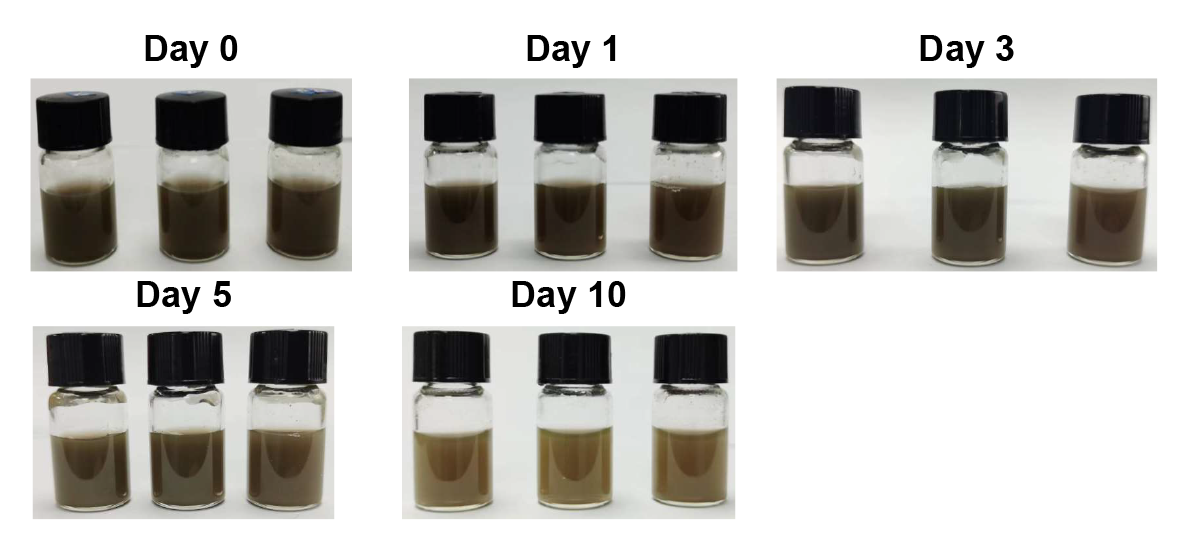
**

**Figure S2.** The stability images of BP, BD and BDM observed at 0, 1, 3, 5 and 10 days.

**Figure S3**

**
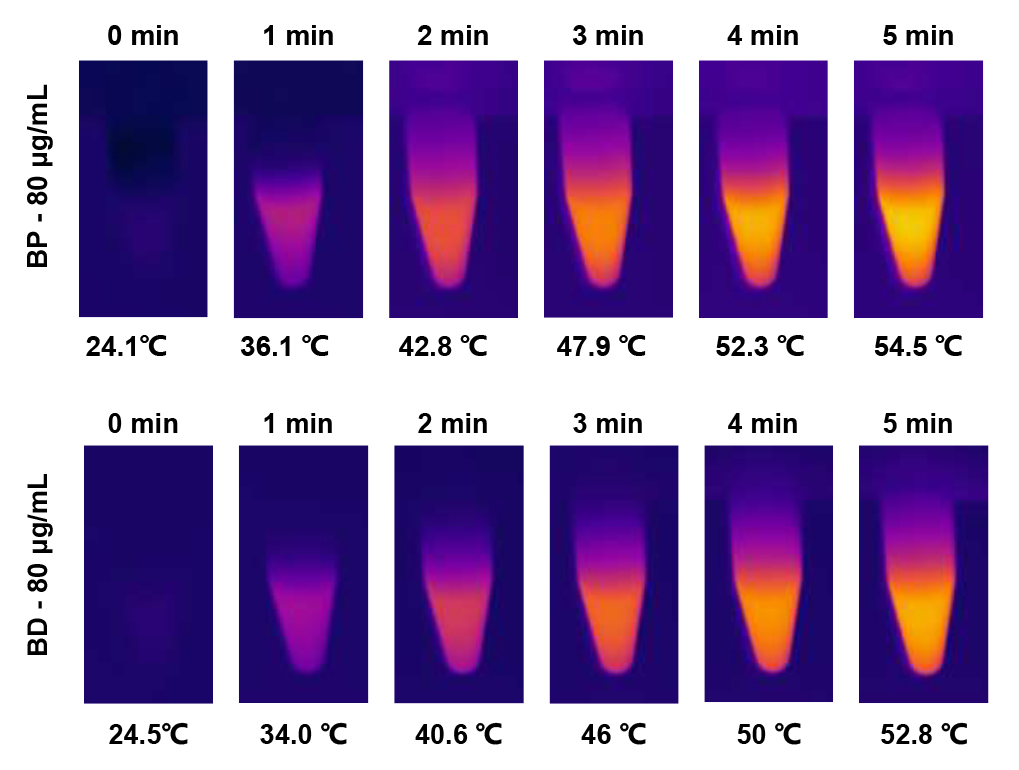
**

**Figure S3.** Photothermal images of BP and BD (80 μg/mL) in different times with laser irradiation (808nm, 1.5 W/cm2).

**Figure S4**

**
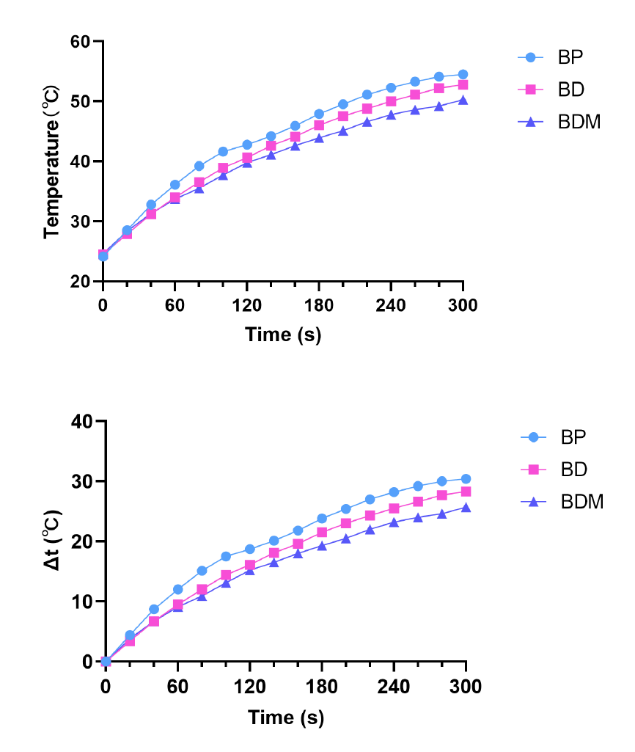
**

**Figure S4.** The change of temperature (Δt) in BP, BD and BDM group.

**Figure S5**

**
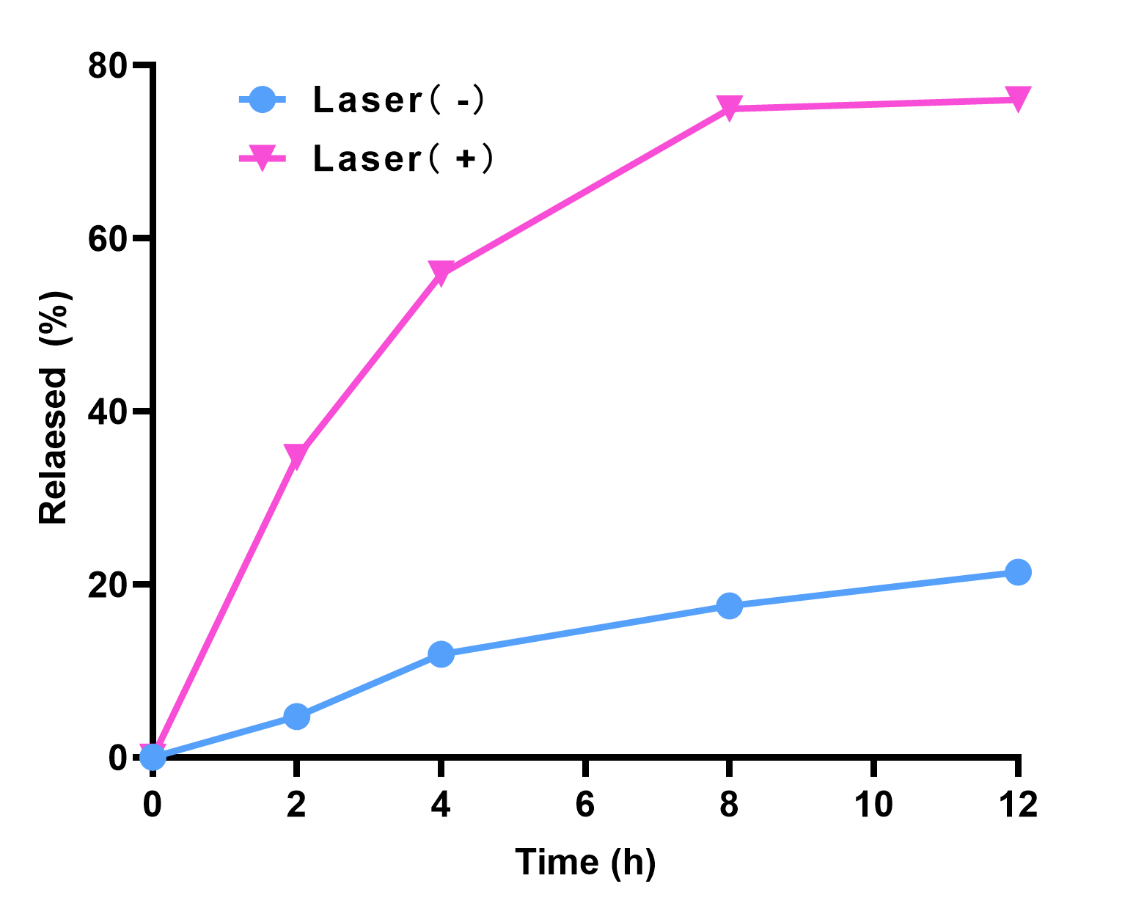
**

**Figure S5.** Decitabine released from BDM at pH 5.0 with or without 808 nm irradiation (1.5 W/cm^2^).

**Figure S6**

**
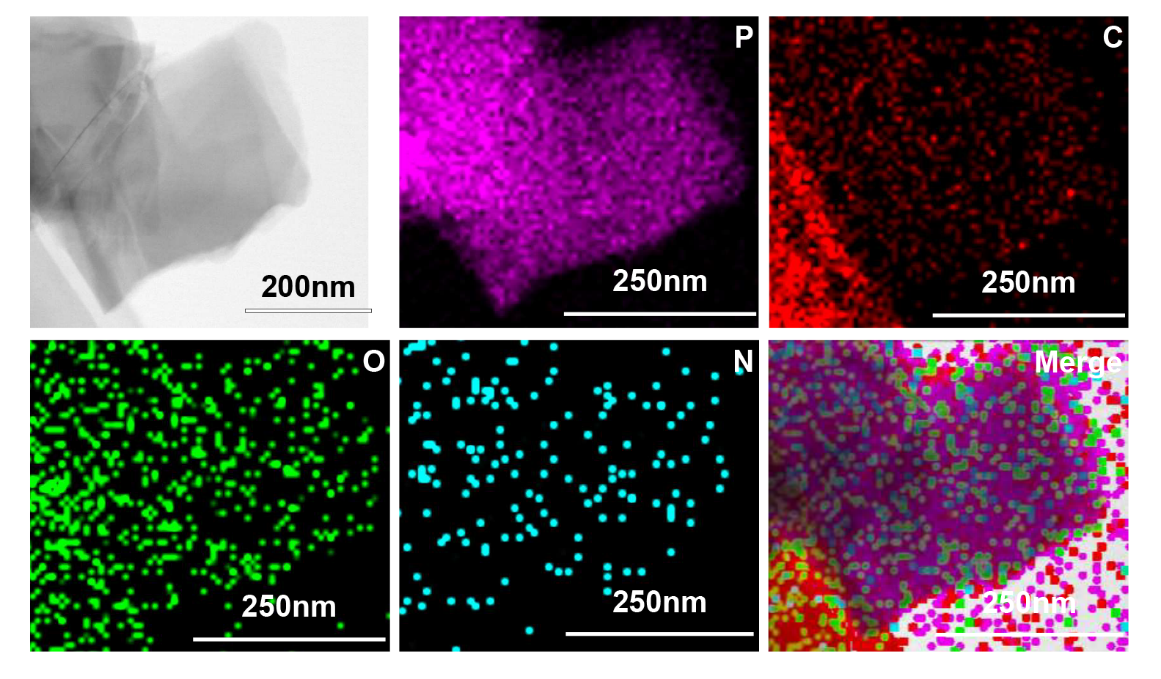
**

**Figure S6.** TEM mapping of BP. Scale bar=200 and 250nm.

**Figure S7**

**
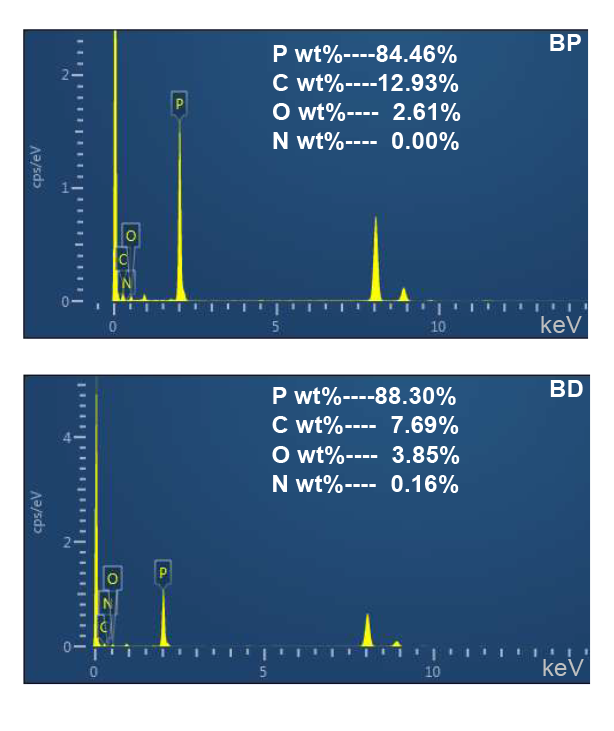
**

**Figure S7.** The Mass fraction of P, C, O and N element in BP and BD group.

**Figure S8**

**
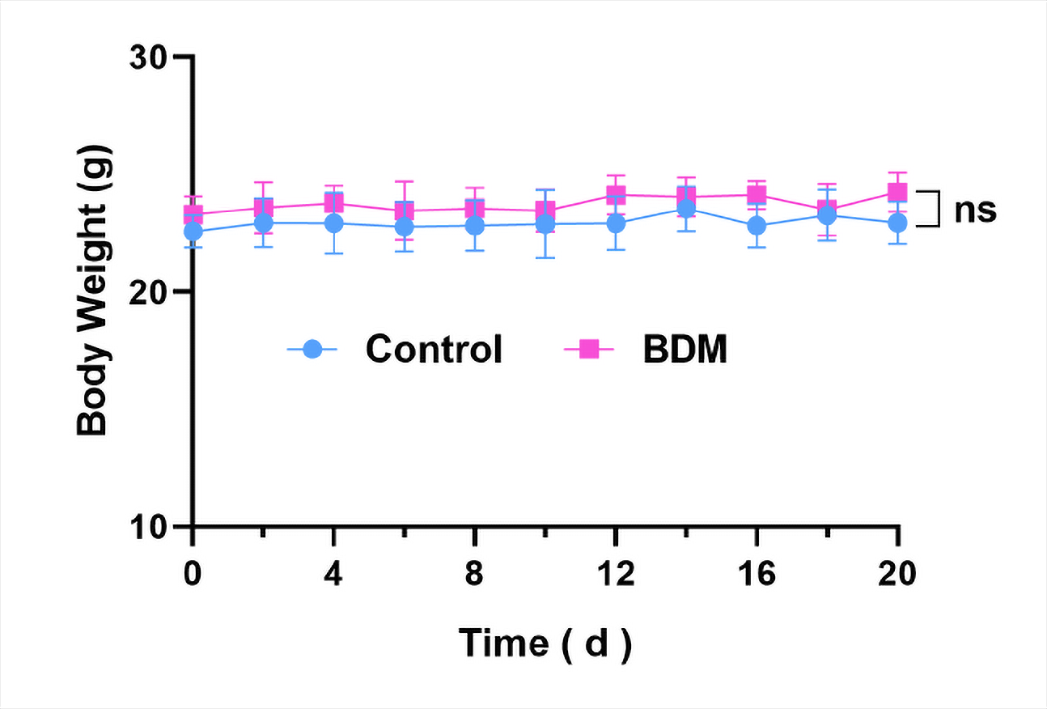
**

**Figure S8.** Systemic toxicity evaluation of BDM: The body weight change of mice with or without BDM treatment (i.v. twice a week, 80 μg/mL, n=3 per group).

**Figure S9**


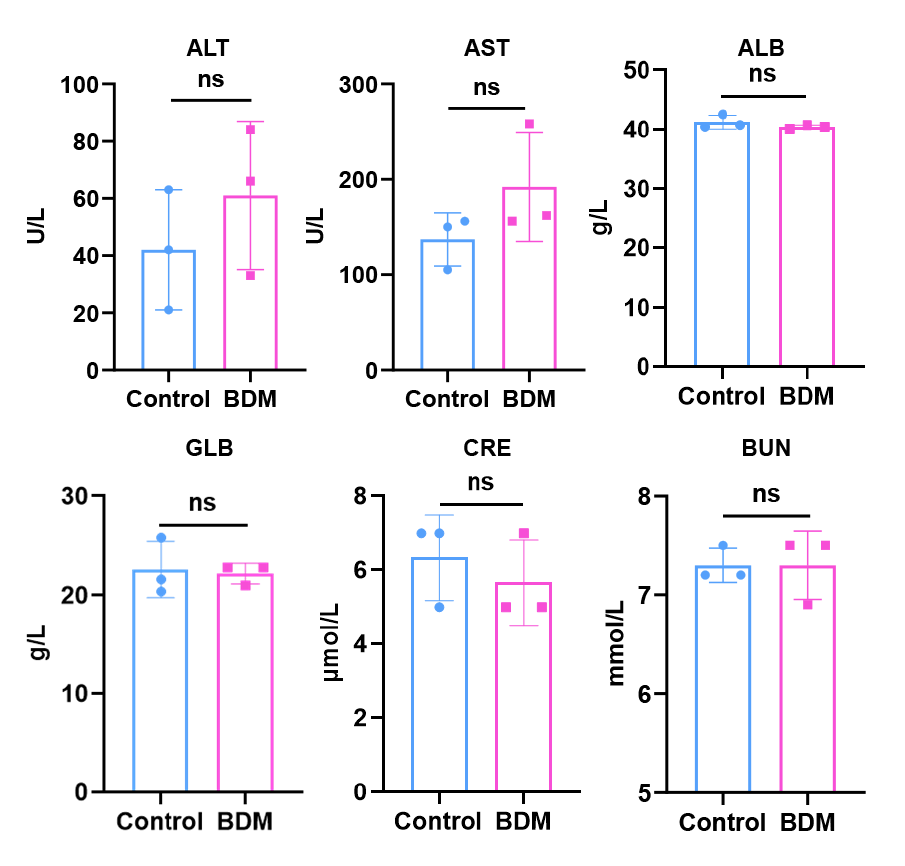


**Figure S9.** Systemic toxicity evaluation of BDM: ALT, ALP, ALB, GLB, CRE and BUN (n=3 per group).

**Figure S10**


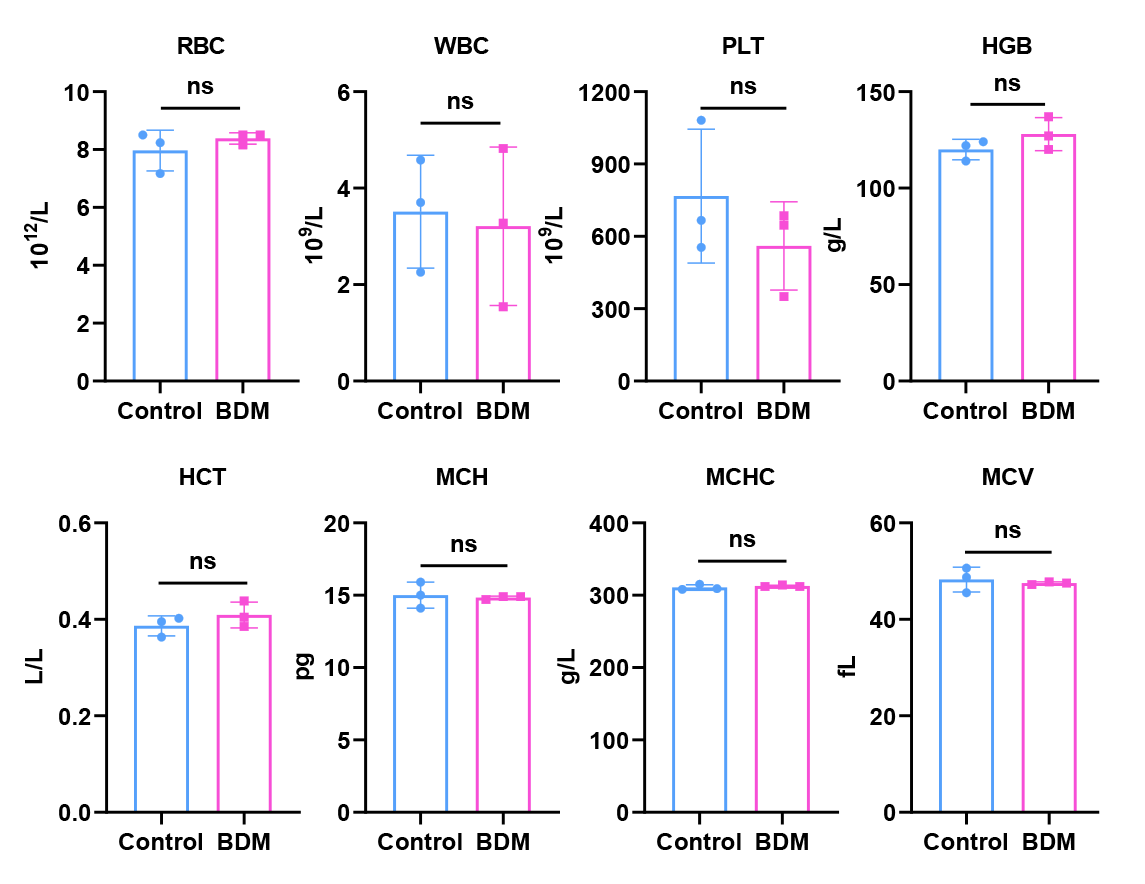


**Figure S10.** Systemic toxicity evaluation of BDM: RBC, WBC, PLT, HGB, HCT, MCH, MCHC and MCV (n=3 per group).

**Figure S11**


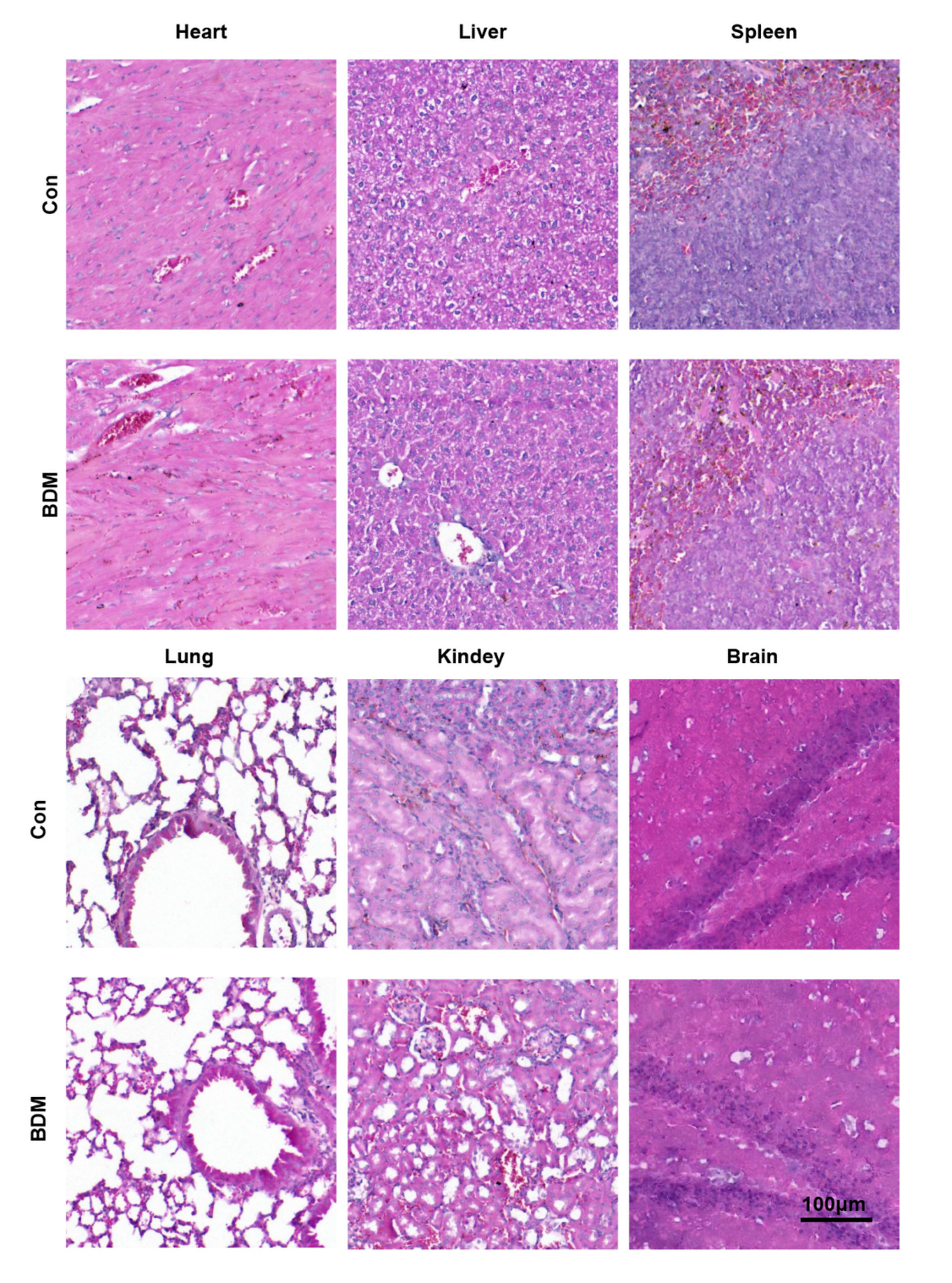


**Figure S11.** Hematoxylin and eosin (HE) staining of organs from mice with or without BDM treatment (n=3 per group). Scale bar: 100 µm.

**Figure S12**


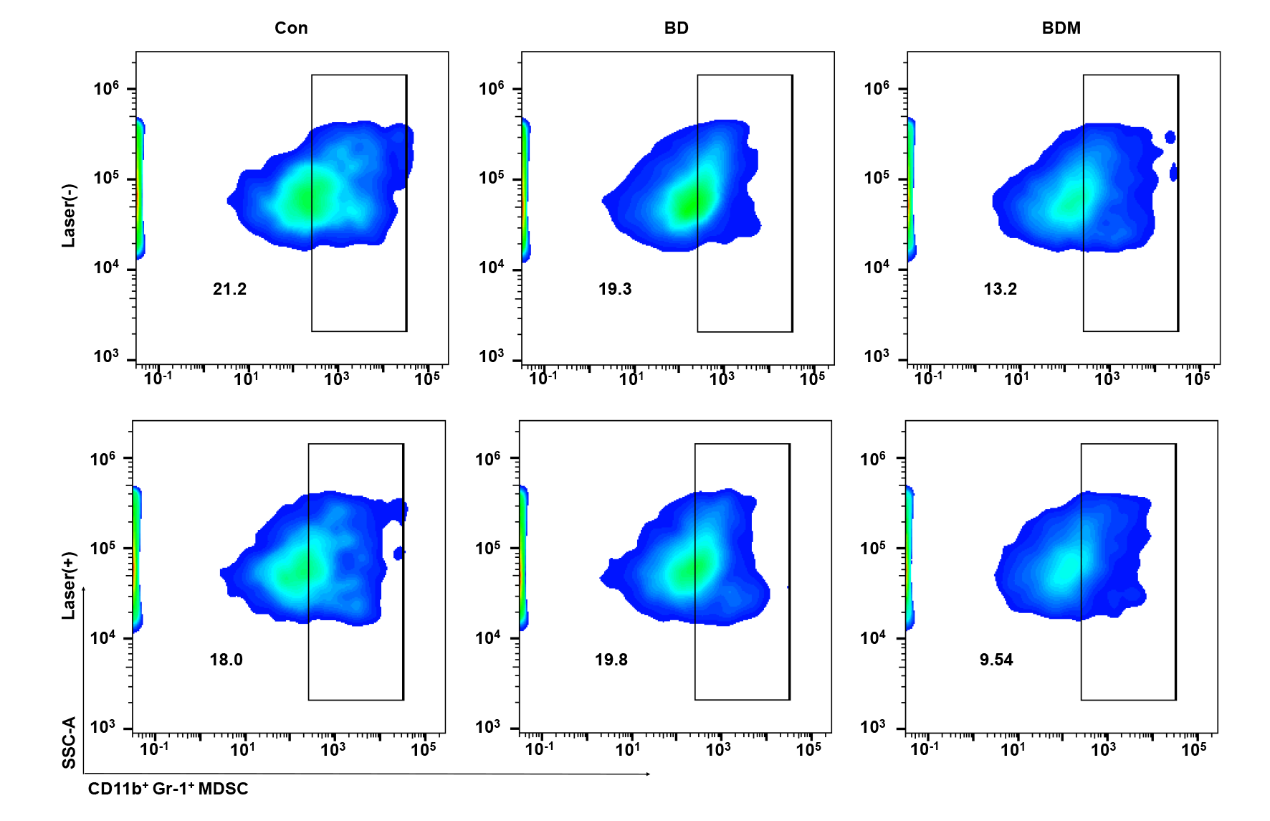


**Figure S12.** Representative flow cytometry data for CD11b^+^ Gr-1^+^ MDSCs in tumor microenvironment of different groups. (n=3 per group, G1: Con, G2: Laser (L), G3: BD, G4: BDM, G5: BD+L, G6: BDM+L).

**Figure S13**


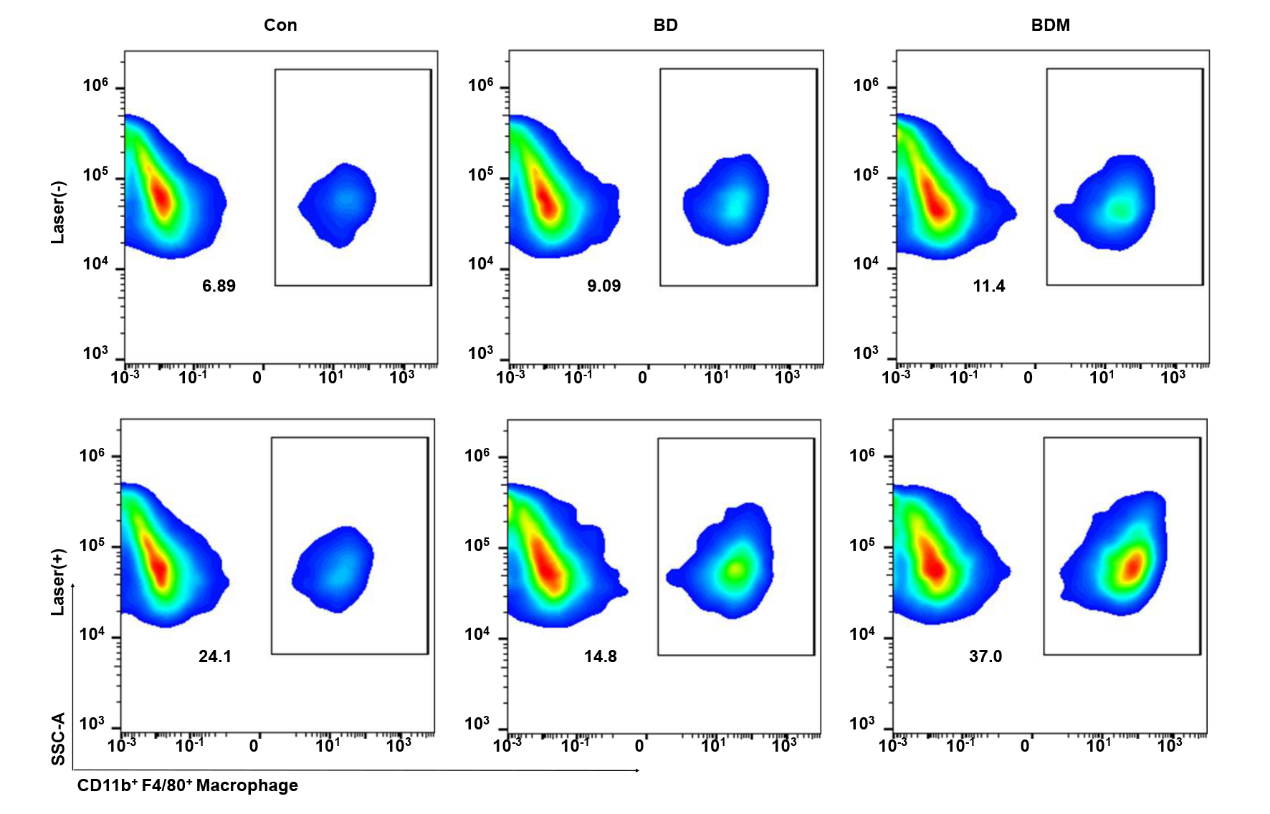


**Figure S13.** Representative flow cytometry data for CD11b^+^ F4/80^+^ Macrophages in tumor microenvironment of different groups. (n=3 per group, G1: Con, G2: Laser (L), G3: BD, G4: BDM, G5: BD+L, G6: BDM+L).

**Figure S14**


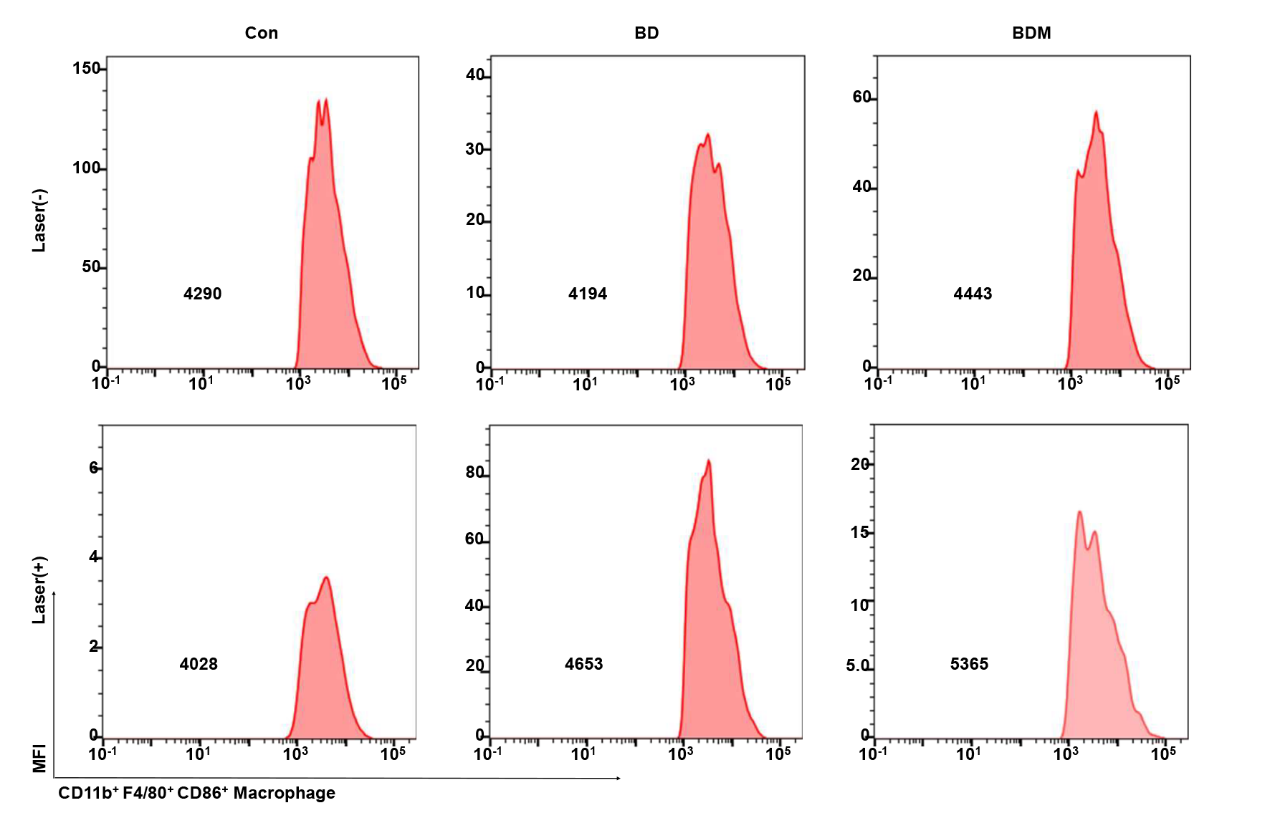


**Figure S14.** Representative flow cytometry data for CD86^+^ CD11b^+^ F4/80^+^ M1-like Macrophages in tumor microenvironment of different groups. (n=3 per group, G1: Con, G2: Laser (L), G3: BD, G4: BDM, G5: BD+L, G6: BDM+L).

**Figure S15**


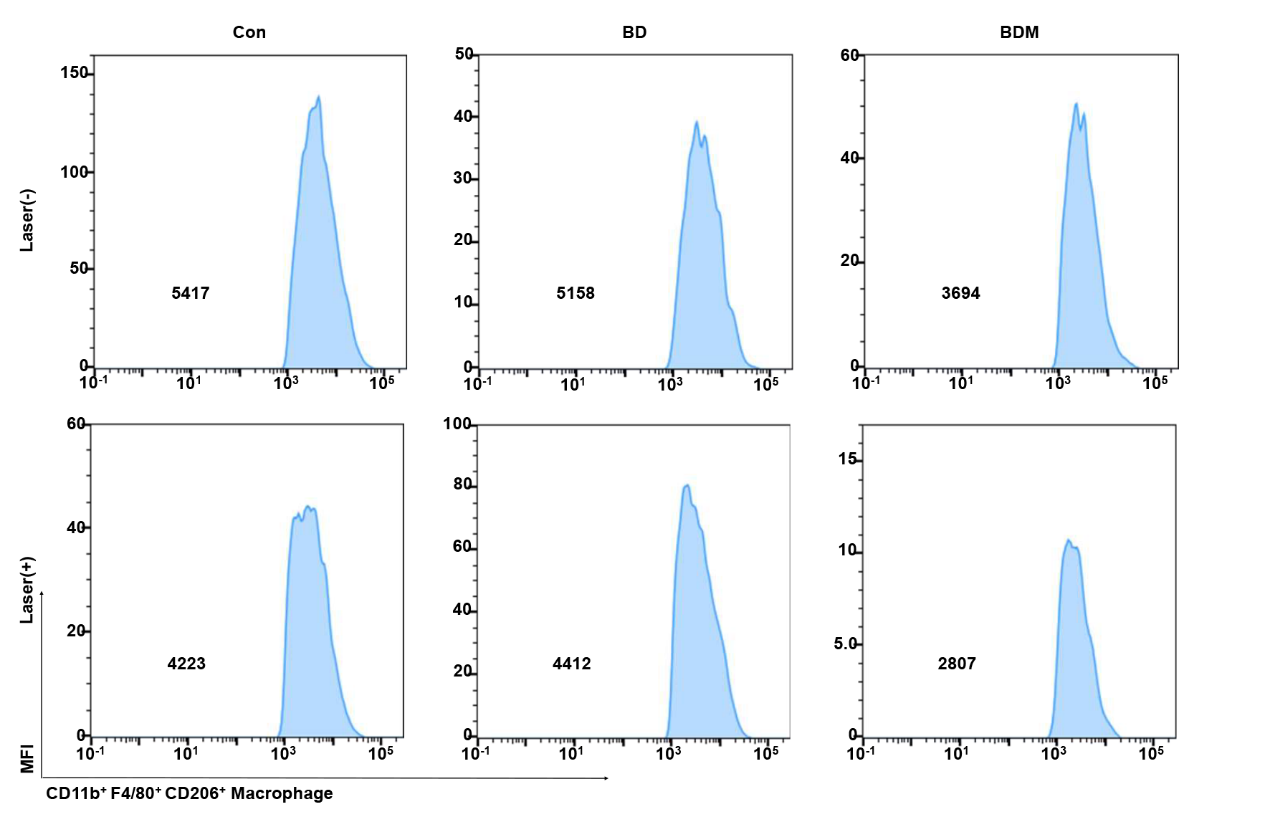


**Figure S15.** Representative flow cytometry data for CD206^+^ CD11b^+^ F4/80^+^ M2-like Macrophages in tumor microenvironment of different groups. (n=3 per group, G1: Con, G2: Laser (L), G3: BD, G4: BDM, G5: BD+L, G6: BDM+L).

**Figure S16**


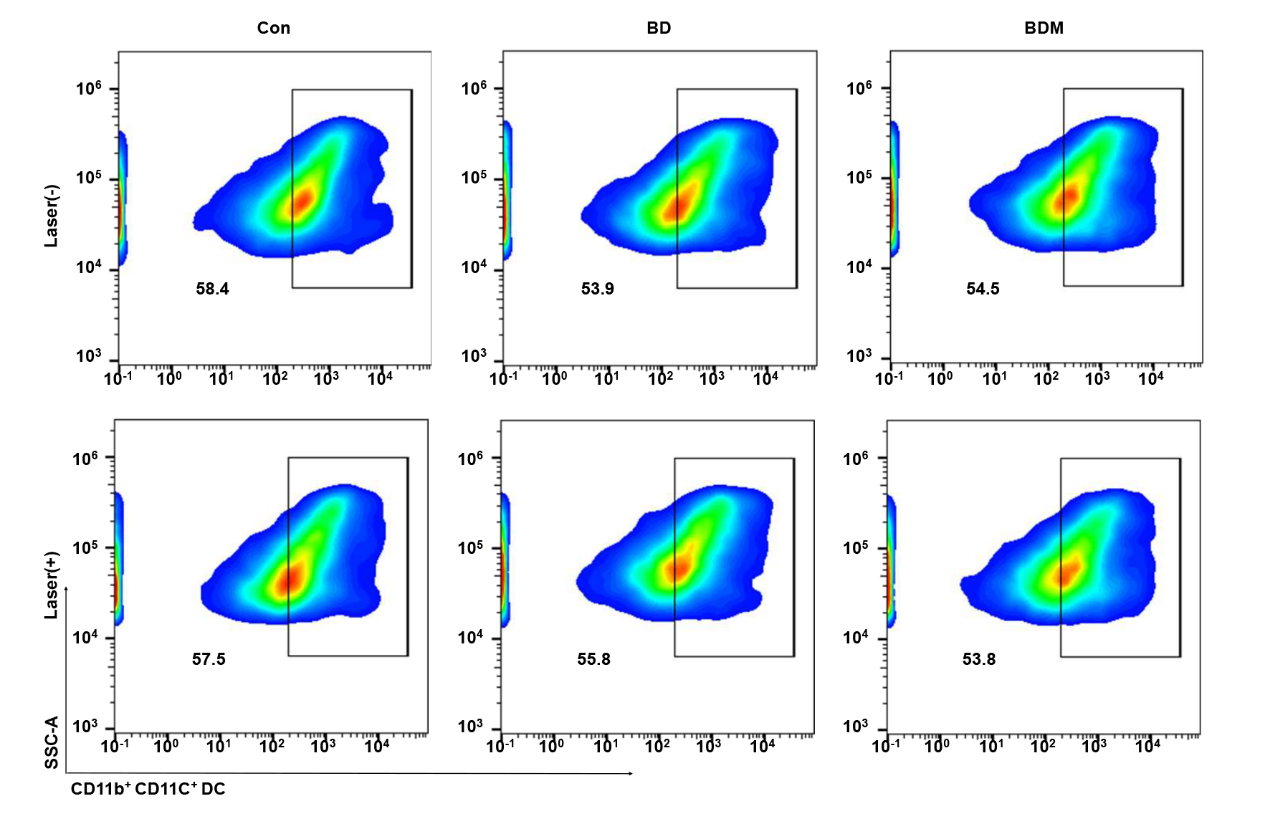


**Figure S16.** Representative flow cytometry data for CD11b^+^ CD11c^+^ DCs in tumor microenvironment of different groups. (n=3 per group, G1: Con, G2: Laser (L), G3: BD, G4: BDM, G5: BD+L, G6: BDM+L).

**Figure S17**


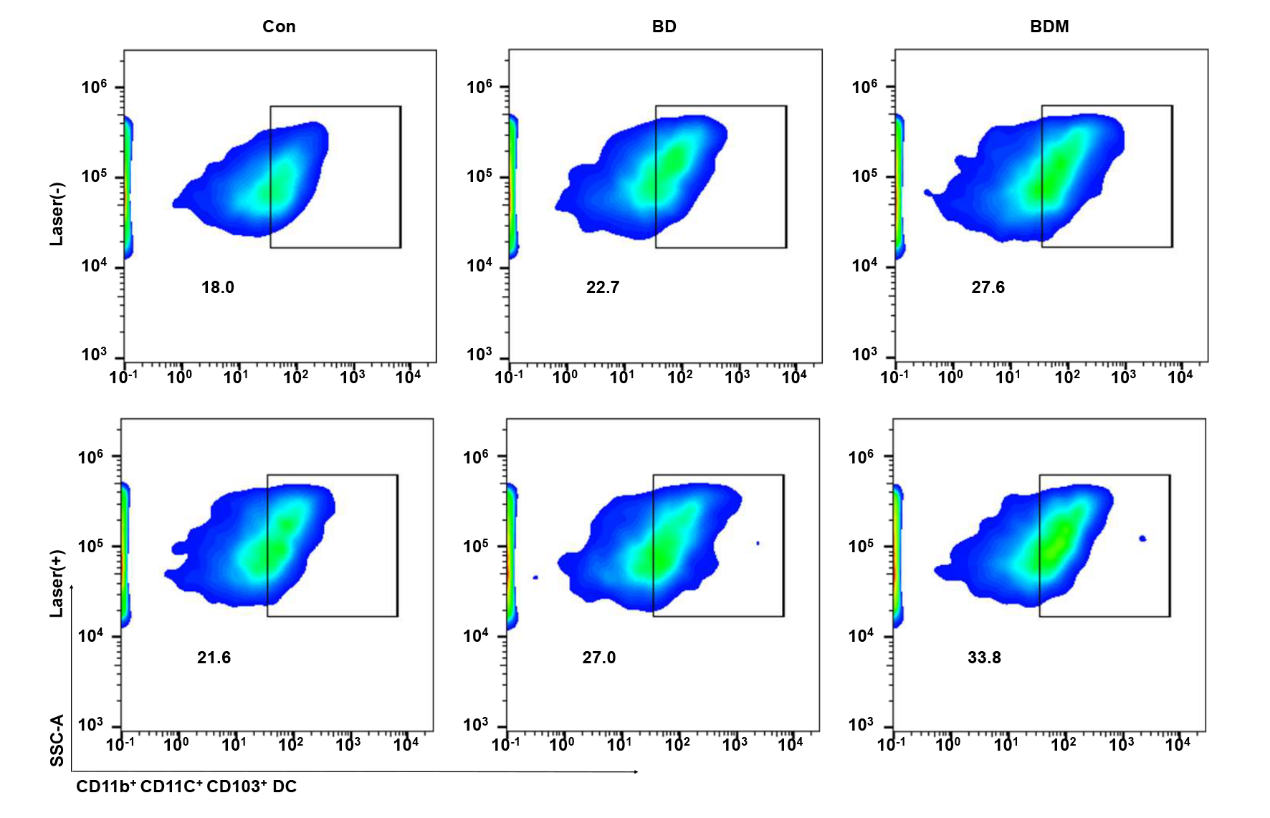


**Figure S17.** Representative flow cytometry data for CD103^+^ CD11b^+^ CD11c^+^ Matured DCs in tumor microenvironment of different groups. (n=3 per group, G1: Con, G2: Laser (L), G3: BD, G4: BDM, G5: BD+L, G6: BDM+L).

**Figure S18**


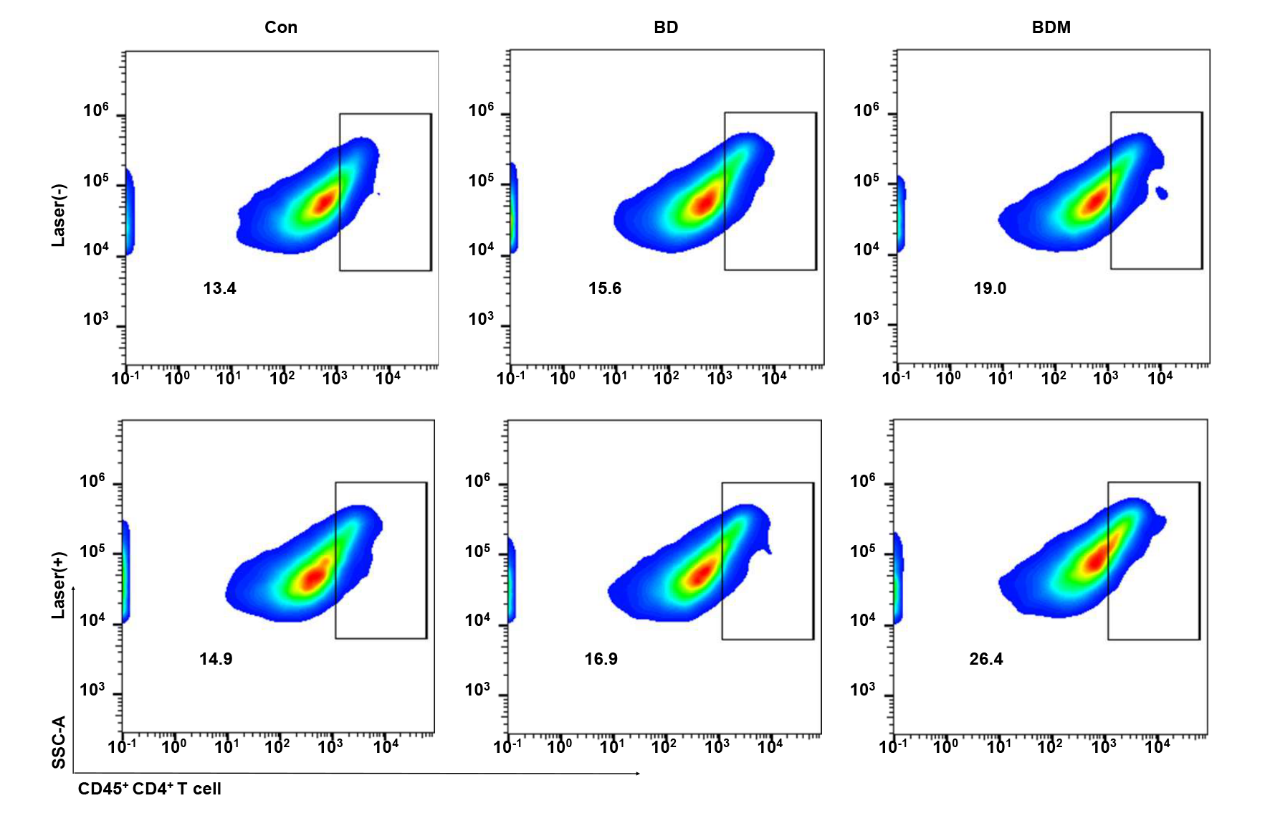


**Figure S18.** Representative flow cytometry data for CD45^+^ CD4^+^ T cells in tumor microenvironment of different groups. (n=3 per group, G1: Con, G2: Laser (L), G3: BD, G4: BDM, G5: BD+L, G6: BDM+L).

**Figure S19**


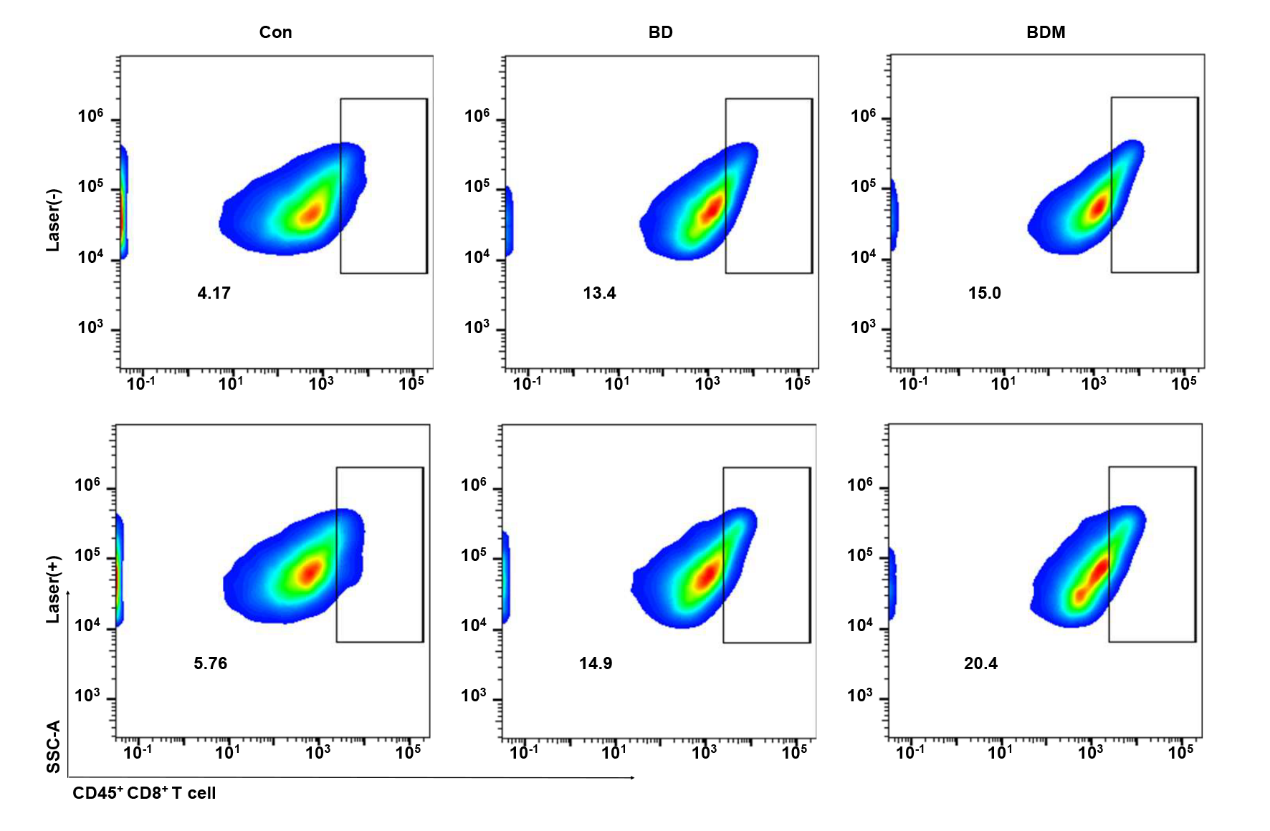


**Figure S19.** Representative flow cytometry data for CD45^+^ CD8^+^ T cells in tumor microenvironment of different groups. (n=3 per group, G1: Con, G2: Laser (L), G3: BD, G4: BDM, G5: BD+L, G6: BDM+L).

**Figure S20**


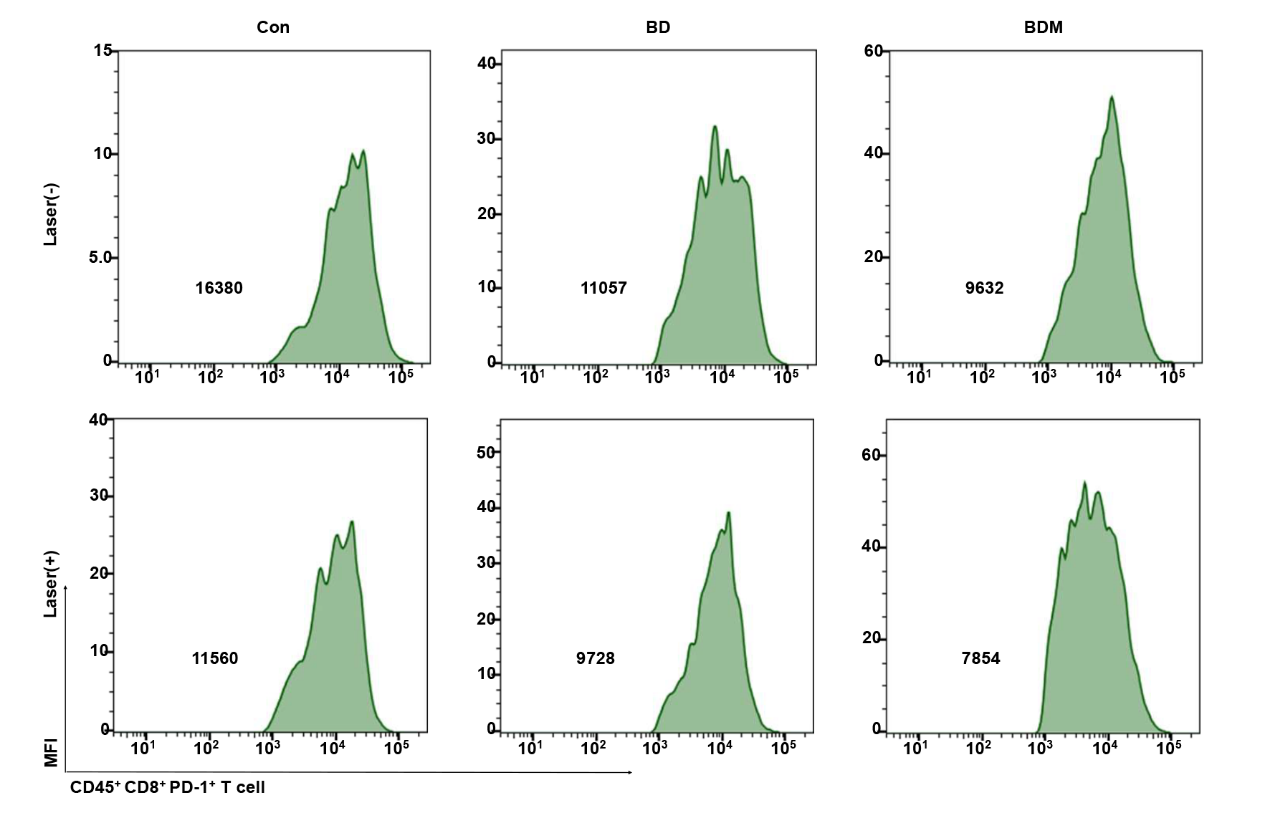


**Figure S20.** Representative flow cytometry data for CD45^+^ CD8^+^ PD-1^+^ T cells in tumor microenvironment of different groups. (n=3 per group, G1: Con, G2: Laser (L), G3: BD, G4: BDM, G5: BD+L, G6: BDM+L).
